# Reduced level of docosahexaenoic acid shifts GPCR neuroreceptors to less ordered membrane regions

**DOI:** 10.1371/journal.pcbi.1007033

**Published:** 2019-05-20

**Authors:** Matti Javanainen, Giray Enkavi, Ramon Guixà-Gonzaléz, Waldemar Kulig, Hector Martinez-Seara, Ilya Levental, Ilpo Vattulainen

**Affiliations:** 1 Computational Physics Laboratory, Tampere University, Tampere, Finland; 2 Department of Physics, University of Helsinki, Helsinki, Finland; 3 Institute of Organic Chemistry and Biochemistry of the Czech Academy of Sciences, Prague, Czech Republic; 4 Laboratory of Computational Medicine, Biostatistics Unit, Faculty of Medicine, Autonomous University of Barcelona, Bellaterra, Spain; 5 Department of Integrated Biology and Pharmacology, McGovern Medical School, University of Texas Health Science Center at Houston, Houston, United States of America; 6 MEMPHYS – Center for Biomembrane Physics; Max Planck Institute for Biophysical Chemistry, GERMANY

## Abstract

G protein-coupled receptors (GPCRs) control cellular signaling and responses. Many of these GPCRs are modulated by cholesterol and polyunsaturated fatty acids (PUFAs) which have been shown to co-exist with saturated lipids in ordered membrane domains. However, the lipid compositions of such domains extracted from the brain cortex tissue of individuals suffering from GPCR-associated neurological disorders show drastically lowered levels of PUFAs. Here, using free energy techniques and multiscale simulations of numerous membrane proteins, we show that the presence of the PUFA DHA helps helical multi-pass proteins such as GPCRs partition into ordered membrane domains. The mechanism is based on hybrid lipids, whose PUFA chains coat the rough protein surface, while the saturated chains face the raft environment, thus minimizing perturbations therein. Our findings suggest that the reduction of GPCR partitioning to their native ordered environments due to PUFA depletion might affect the function of these receptors in numerous neurodegenerative diseases, where the membrane PUFA levels in the brain are decreased. We hope that this work inspires experimental studies on the connection between membrane PUFA levels and GPCR signaling.

## Author summary

Our current picture of cellular membranes depicts them as laterally heterogeneous sheets of lipids crowded with membrane proteins. These proteins often require a specific lipid environment to efficiently perform their functions. Certain neuroreceptor proteins are regulated by membrane cholesterol that is considered to be enriched in ordered membrane domains. In the brain, these very same domains also contain a fair amount of polyunsaturated fatty acids (PUFAs) that have also been discovered to interact favorably with many receptor proteins. However, certain neurological diseases—associated with the inadequate functioning of the neuroreceptors—seem to result in the decrease of brain PUFA levels. We hypothesized that this decrease in PUFA levels somehow inhibits receptor partitioning to cholesterol-rich domains, which could further compromise their function. We verified our hypothesis by an extensive set of computer simulations. They demonstrated that the PUFA–receptor interaction indeed leads to favorable partitioning of the receptors in the cholesterol-rich ordered domains. Moreover, the underlying mechanism based on the shielding of the rough protein surface by the PUFAs seems to be exclusive for multi-helical protein structures, of which neuroreceptors are a prime example.

## Introduction

Cellular membranes host functional membrane domains (“lipid rafts’’) rich in proteins and cholesterol (CHOL) [1]. Many G protein-coupled receptors (GPCRs) and cognate G proteins are found in these domains [2], and numerous reports have suggested that CHOL is involved in GPCR function [3–7]. Moreover, impaired CHOL homoeostasis and raft disruption have been linked to different neurodegenerative diseases [2, 8], where GPCRs play a pivotal role. However, the mechanism driving the partitioning of GPCRs to their native functional CHOL-rich environments is still not well understood.

Polyunsaturated fatty acids (PUFAs) such as docosahexaenoic acid (DHA, 22:6(n-3)) are likewise key membrane components of brain cells [9]. PUFAs esterify to phospholipids together with a saturated chain to form a *hybrid lipid*. Intriguingly, despite their disordered nature, hybrid lipids are found in raft extracts [10–12], and they also partition surprisingly well to cholesterol-rich ordered membrane regions [13]. However, raft PUFA levels are reduced in various neuropsychiatric and mental disorders [14] including Alzheimer’s [10] and Parkinson’s diseases [11]. This lack of PUFAs could thus affect GPCR function. In fact, experiments have shown that DHA-containing lipids enhance the function of the prototypical GPCR rhodopsin [15–17], which simulation studies have explained to take place as a result of the high conformational flexibility of DHA chains. This provides hybrid lipids with high affinity for the rough surface of GPCRs, [18–21] further promoting protein–protein interactions [22].

We recently reported the high affinity of DHA for the adenosine A_2A_ receptor (A_2A_R) [23], a GPCR with an important role in the central nervous systems, where different antagonists of A_2A_R have shown promising neuroprotective effects [24, 25]. Membrane CHOL is also known to closely interact with A_2A_R [7, 26–28], modulating its function [29] and ligand binding properties [7]. The partitioning of A_2A_R into ordered membrane domains [30] is therefore quite expected, though the mechanism rendering it possible has been suggested to be complex [31]. Moreover, given the numerous factors affecting protein partitioning [32] and the limited ability of model systems to capture *in vivo* behavior [33], it is not surprising that the role of PUFAs in A_2A_R partitioning remains to be investigated. Given the central role of GPCRs in cell signaling, unlocking how DHA interacts with GPCRs is the key to understanding why GPCR function is impaired in severe brain diseases associated with a lowered membrane DHA level.

Here, we studied the role of PUFAs in the partitioning of GPCRs into CHOL-rich (raft-like) liquid-ordered (L_o_) and CHOL-depleted liquid-disordered (L_d_) phases. Combining all-atom and coarse-grained molecular dynamics (MD) simulations with free energy calculations, we demonstrate for A_2A_R that in the absence of DHA, corresponding to brain tissue of diseased individuals, partitioning to the L_d_ phase is energetically favored. However, in membranes including DHA-containing hybrid lipids, corresponding to brain tissue of healthy individuals, DHA drives A_2A_R to partition to the L_o_ phase, as a favorable structural arrangement of DHA around A_2A_R minimizes the structural perturbations therein. Furthermore, based on our studies on a number of distinct membrane proteins, we demonstrate that the observed effect of DHA could be limited to rough helical multi-pass membrane proteins, which include GPCRs.

## Results

### DHA promotes A_2A_R partitioning into the ordered phase

**Fig 1:**
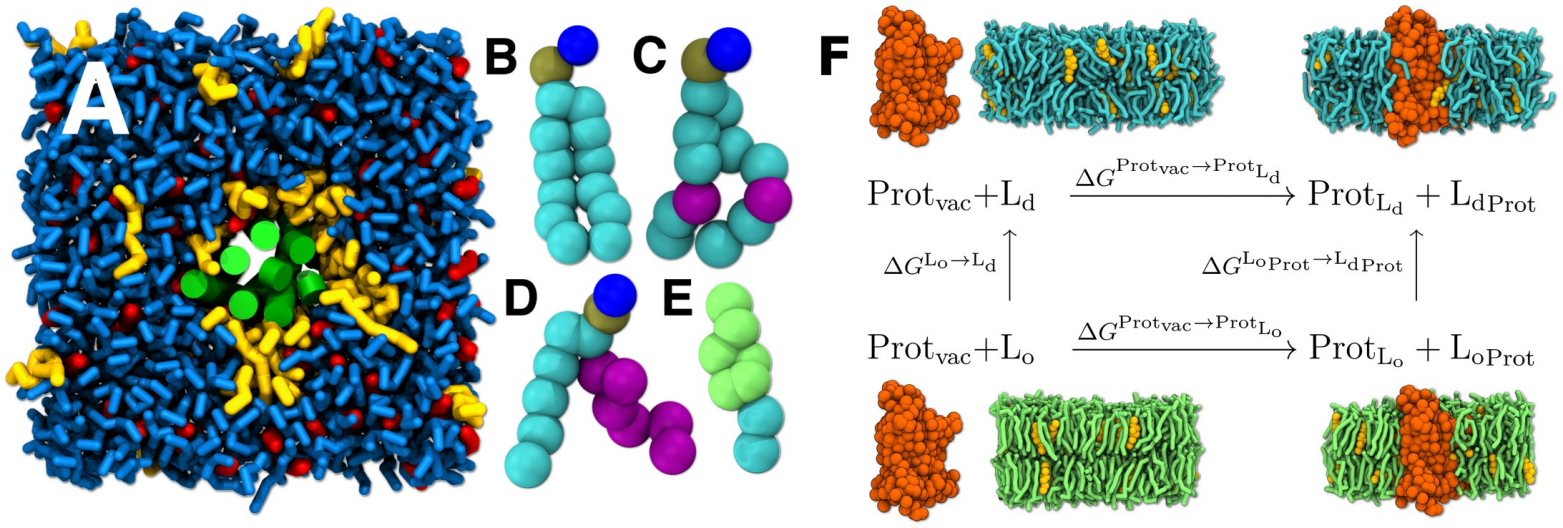
A snapshot of a simulated membrane, the used lipid moieties, and the thermodynamic cycle for estimation of the free energy of transfer of a membrane protein between the L_d_ and L_o_ phases. A) L_o_ membrane with 8 mol% SDPE after 10 μs of equilibration. In panel A), A_2A_R is shown in green, DSPC in blue, CHOL in red, and SDPE in yellow. CG structures of B) DSPC, C) DOPC, D) SDPE, and E) CHOL. In panels B)–E), phosphate is shown in brown, choline/ethanolamine in blue, and the CHOL ring in green. The saturated chain segments and glycerol are shown in cyan, while the unsaturated chains are shown in purple. F) The thermodynamic cycle. The horizontal arrows represent the transformations used in this work, whereas the vertical arrows represent the alternative transformations employed commonly for smaller molecules (see Section A.1 in S1 File for details). Lipids having a low main transition temperature *T*_m_ (here DOPC, present in the L_d_ phase) and lipids having a high *T*_m_ (here DSPC, present in the L_o_ phase) are shown in cyan and green, respectively. Cholesterol is shown in yellow and the protein in orange.

**Fig 2:**
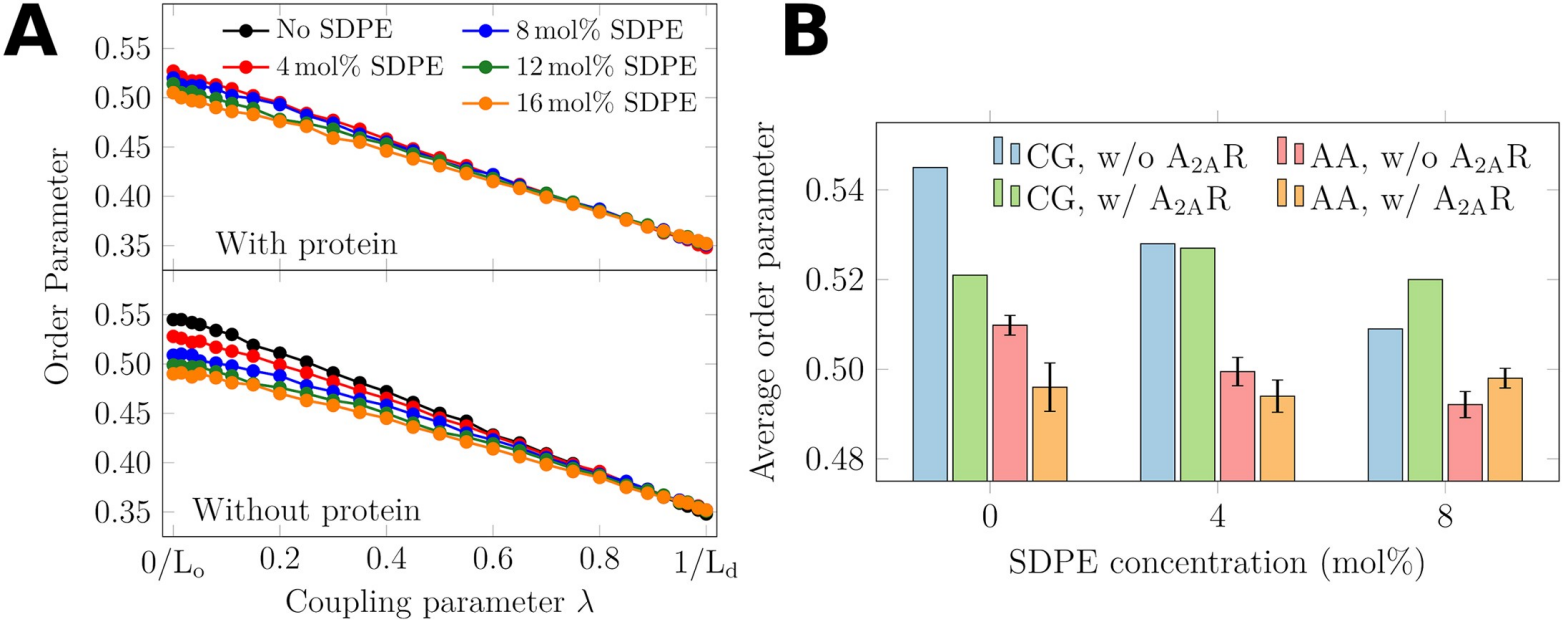
Average 2^nd^ rank acyl chain order parameters (*S*_*n*_). A) Average order parameter of the mutated lipid (DSPC→DOPC) in (top) the system containing A_2A_R, and (bottom) the protein-free system. The cholesterol content was 20 mol% in all systems. The L_o_→L_d_ transformation takes place as the coupling parameter λ changes from zero to one. In Section B.1 in the S1 File, we demonstrate that these end points indeed correspond to the L_o_ and L_d_ phases. Error bars showing standard error are smaller than the marker size. B) Effects of SDPE and A_2A_R on average 2^nd^ rank acyl chain order parameters ($\bar{S_{n}}$). Data are shown for both coarse-grained and all-atom (fine-grained) simulations. For the latter, the order parameter is estimated from the average deuterium order parameter as $\bar{S_{n}}=-2\bar{S_{\text{CD}}}$, and the error bars show the standard error.

We calculated the free energy of transfer of A_2A_R between L_o_ and L_d_ phases in the coarse-grained (CG) scheme using the non-polarizable Martini 2.2 model [34–36]. First, we embedded the protein in an L_o_ phase membrane containing distearoylphosphatidylcholine (DSPC, Fig 1B), 20 mol% CHOL (Fig 1E), and different concentrations of stearoyldocosahexaenoylphosphatidylethanolamine (SDPE, Fig 1D) with a polyunsaturated DHA chain, see Fig 1A. In line with lipidomics experiments, DHA was paired with the PE head group. [37] Next, we mutated L_o_-forming DSPC to L_d_-forming dioleoylphosphatidylcholine (DOPC, Fig 1C) in a set of simulations and extracted the free energy change $\Delta G^{{\text{L}}_{{\text{o}}_{\text{Prot}}}\to {\text{L}}_{{\text{d}}_{\text{Prot}}}}$ using the free energy perturbation approach. Here, a coupling parameter λ has a value of 0 for DSPC and 1 for DOPC. Then, we obtained $\Delta G^{{\text{L}}_{\text{o}}\to {\text{L}}_{\text{d}}}$ by repeating this calculation in the absence of the protein. As discussed in Section B.7 in the S1 File, it is possible that the experimentally observed microscopic phase separation in this DOPC/DSPC/CHOL mixture [38] is associated by a fairly large line tension and hence only takes place in membranes larger than those currently within the reach of MD simulations. This limits us from studying protein partitioning in DOPC/DSPC/CHOL mixtures with coexisting domains. Nevertheless, the lipid chain order parameters, shown in Fig 2A as a function of λ, demonstrate a smooth transition between distinct L_o_ and L_d_ phases in both sets of the simulations. We therefore believe that our approach is able to capture the physical properties of the coexisting phases in isolation. Further analyses shown in Section B.1 in the S1 File also support this view. Following the thermodynamic cycle depicted in Fig 1F, we carried on to extract the free energies of transfer as ($\Delta G^{{\text{L}}_{{\text{o}}_{\text{Prot}}}\to {\text{L}}_{{\text{d}}_{\text{Prot}}}}-\Delta G^{{\text{L}}_{\text{o}}\to {\text{L}}_{\text{d}}}$). Additionally, we also used a more realistic composition—based on the tie lines measured for the DOPC/DPPC/CHOL mixture—where the DSPC/DOPC ratio was 2.3 in the L_o_ phase, and then reversed to 1/2.3 in the L_d_ phase. For further details on our computational approach, the system compositions, and the simulation parameters, see Methods and the S1 File.

**Fig 3:**
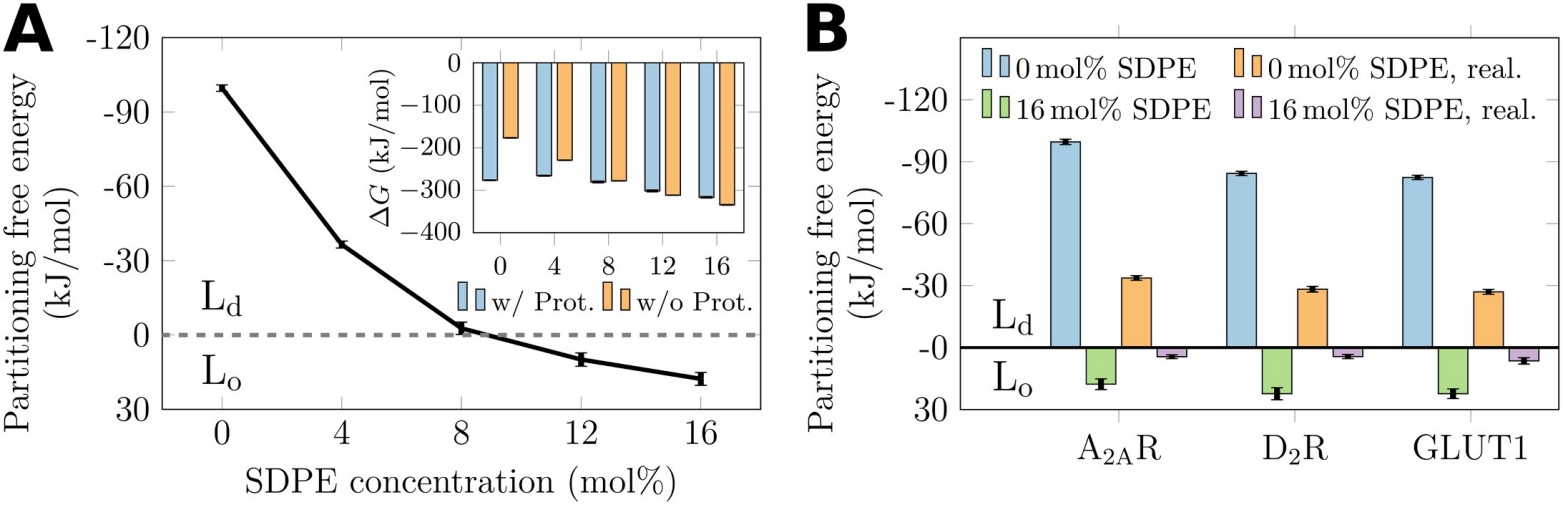
The effect of SDPE on the free energies of transfer of proteins between the L_o_ and L_d_ phases. A) Values for A_2A_R are shown in black solid line. A negative sign of the free energy of transfer indicates A_2A_R to favor the L_d_ phase. Inset shows the free energy changes $\Delta G^{{\text{L}}_{{\text{o}}_{\text{Prot}}}\to {\text{L}}_{{\text{d}}_{\text{Prot}}}}$ and $\Delta G^{{\text{L}}_{\text{o}}\to {\text{L}}_{\text{d}}}$ of the alchemical transformations (DSPC→DOPC) with and without A_2A_R, respectively. B) Free energies of all studied proteins in the absence and presence (16 mol%) of SDPE. The more realistic membrane compositions are denoted with “real.”.

The free energy of transfer of A_2A_R as a function of SDPE concentration is shown as a solid line in Fig 3A. Strikingly, the free energy of transfer changes sign at the SDPE concentration of ∼8 mol%. This highlights that for dilute concentrations of SDPE, A_2A_R partitions to the L_d_ phase. However, at higher SDPE concentrations the picture changes completely and the protein favors partitioning to the L_o_ phase.

Concluding, the data provide compelling evidence that the presence of SDPE, and therefore DHA, makes A_2A_R compatible with the L_o_ phase.

### A_2A_R surface is saturated with DHA

**Fig 4:**
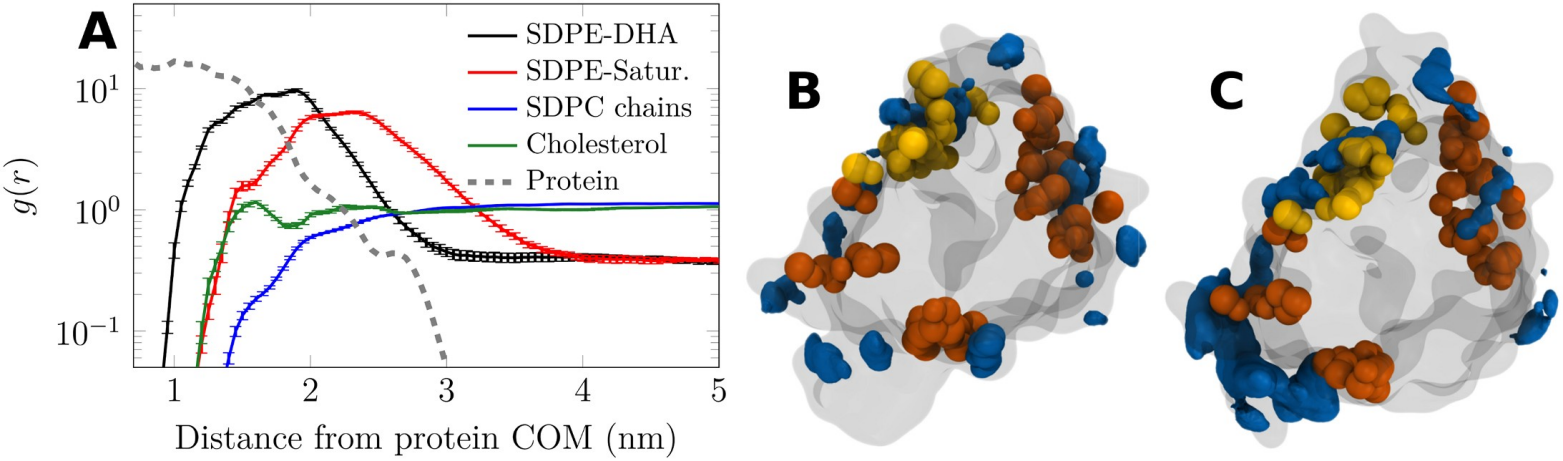
2D radial distribution functions of different lipid chains around the center of mass of A_2A_R and cholesterol density maps. A) The radial distribution functions are shown as a function of distance from the A_2A_R COM for the L_o_ phase membrane (DSPC/CHOL) with 4 mol% SDPE. Data are extracted from the last 5 μs of the 10 μs simulation using the gmx rdf tool. Error bars show standard error. Data for other SDPE concentrations are shown in Fig. E in the S1 File. B & C) Cholesterol density around A_2A_R in systems B) without SDPE and C) with 16 mol% SDPE is shown as a blue isosurface. The residues involved in binding cholesterol, suggested by earlier studies, are shown in orange [27] and yellow [28]. The vicinity of these residues to the observed cholesterol density (blue) suggests that cholesterol reaches the protein surface and often finds the proper binding sites in our simulations. Note that the isosurfaces are not in scale.

Fig 4A shows the 2D radial distribution functions (RDFs) of all lipid chain types around A_2A_R in the L_o_ phase with 4 mol% of SDPE. The data are extracted from well-equilibrated membranes in the CG scheme. Fig 4A demonstrates that A_2A_R is fully coated by SDPE with polyunsaturated DHA forming the first solvation shell, followed by the saturated acyl chain of SDPE and CHOL in the second shell. The formation of these shells is illustrated in the movie at DOI:10.6084/m9.figshare.5903881. With increasing SDPE concentration, the right tail of the RDF peaks of all lipids extends further away from the protein (see Fig. E in the S1 File), indicating that the A_2A_R surface becomes saturated with DHA.

Interestingly, Fig 4A shows that CHOL penetrates the shell formed by the saturated chains of SDPE, and occupies annular binding sites, in line with experimental and computational studies on CHOL–A_2A_R interaction [26–28]. Indeed, cholesterol finds the suggested binding sites in the absence (Fig 4B) but also in the presence of (Fig 4C) an SDPE shell.

These lipid shells around A_2A_R are dynamic. Lipids exchange in the time scale of ∼100 ns, as evidenced by the decay time constants found through double exponential fits to the contact data, shown in Table B in the S1 File (see also Section B.2 in the S1 File). For the L_o_ phase, the rates of SDPE and CHOL exchange increase as SDPE concentration increases. In the L_d_ phase, the SDPE corona dissolves (see Fig. G in the S1 File). This lack of a tightly-bound SDPE shell leads to higher SDPE and CHOL exchange rates. Similarly, CHOL exchange rates are also higher in the absence of SPDE. These findings demonstrate that the formation of an SDPE shell also affects the dynamics of CHOL association by stabilizing the neighborhood of A_2A_R.

Concluding, the strong affinity of DHA to interact with A_2A_R leads to coating of the protein by SDPE lipids. DHA is in contact with the protein, whereas the saturated chains favor interactions with CHOL.

### DHA corona renders A_2A_R more compatible with the L_o_ phase

Partitioning of a membrane protein to either the L_o_ or the L_d_ phase is driven by the mutual structural compatibility between the protein and the lipids forming the membrane phase. Possible parameters describing this compatibility include hydrophobic mismatch, the conformational entropy of the protein, and perturbation of lipid chain order. We evaluated the contribution of all these factors in the CG scheme.

Membrane thickness is shown in Fig. K in the S1 File as a function of distance from protein surface. The presence of SDPE has a clear effect on the thickness. Based on the mattress model [39] and using the hydrophobic mismatch parameter from Ref. [32] and the hydrophobic thickness of A_2A_R from the OPM database [40], we estimate that hydrophobic mismatch contributes to the free energy of transfer by approximately 1.8 kJ/mol, favoring the L_d_ phase. However, the presence of 8 mol% of SDPE has an insignificant effect on this value, indicating that negating hydrophobic mismatch is not the mechanism through which SDPE shifts partitioning of A_2A_R towards the L_o_ phase. Notably, this conclusion is insensitive to the value of the hydrophobic mismatch parameter, which might be different between experiment and our simulation model.

Next, we evaluated whether the SDPE corona promotes protein flexibility, hence resulting in a favorable entropic contribution for partitioning to the L_o_ phase in the presence of SDPE. We plot the residue-wise root mean squared fluctuations (RMSF) of the protein structure in both the L_o_ and L_d_ phases in Fig. L in the S1 File. Curiously, in the absence of SDPE, the average RMSF value is slightly higher in the L_o_ phase. However, at 8 mol% of SDPE the average RMSF becomes larger in the L_d_ phase than in the L_o_ phase (see inset in Fig. L in the S1 File). This suggests that the entropic contribution due to the presence of SDPE actually promotes A_2A_R partitioning to the L_d_ phase and hence acts against the observed effect of SDPE on the free energy of transfer. Moreover, we note that the omitted lipid entropies also likely play a role on partitioning.

How about protein-induced changes in lipid acyl chain order? The outer layer of the SDPE corona around A_2A_R is formed by the saturated stearic acid chains of SDPE (see Fig 4A). This layer is likely more compatible with the L_o_ phase than the rough surface of A_2A_R. This idea is indeed backed up by results from CG systems, which show that the effects of SDPE on membrane properties are reduced in the presence of A_2A_R and *vice versa* (see Fig 2A).

We note here that the CG approach is not well-suited to fully characterize acyl chain order. Therefore, we also studied the effects of SDPE and A_2A_R on membrane order in all-atom detail. To this end, we fine-grained selected coarse-grained L_o_ phase systems and carried out all-atom simulations using the CHARMM36 force field [41, 42] as described in Methods. The averaged stearic acid chain order parameters from both all-atom and coarse-grained simulations of the L_o_ phase membranes are shown in Fig 2B.

It is evident that both A_2A_R and SDPE lower the average order of the membrane. However, at an SDPE concentration of 8 mol%, the presence of A_2A_R actually increases membrane order, and this observation holds for both all-atom and coarse-grained schemes. The explanation to this behavior is that when both SDPE and A_2A_R are present, the DHA–A_2A_R interactions shield the order-lowering effects of both SDPE and A_2A_R. Importantly, the values from coarse-grained and atomistic simulations are in the same ballpark. The spatial variation of membrane order due to the presence of A_2A_R is studied in detail in Section B.3 in S1 File.

To conclude, in the L_o_ phase, the association of the flexible DHA chains and the rough surface of A_2A_R weakens their perturbations on membrane (acyl chain) order.

### DHA does not solvate other protein types efficiently

**Fig 5:**
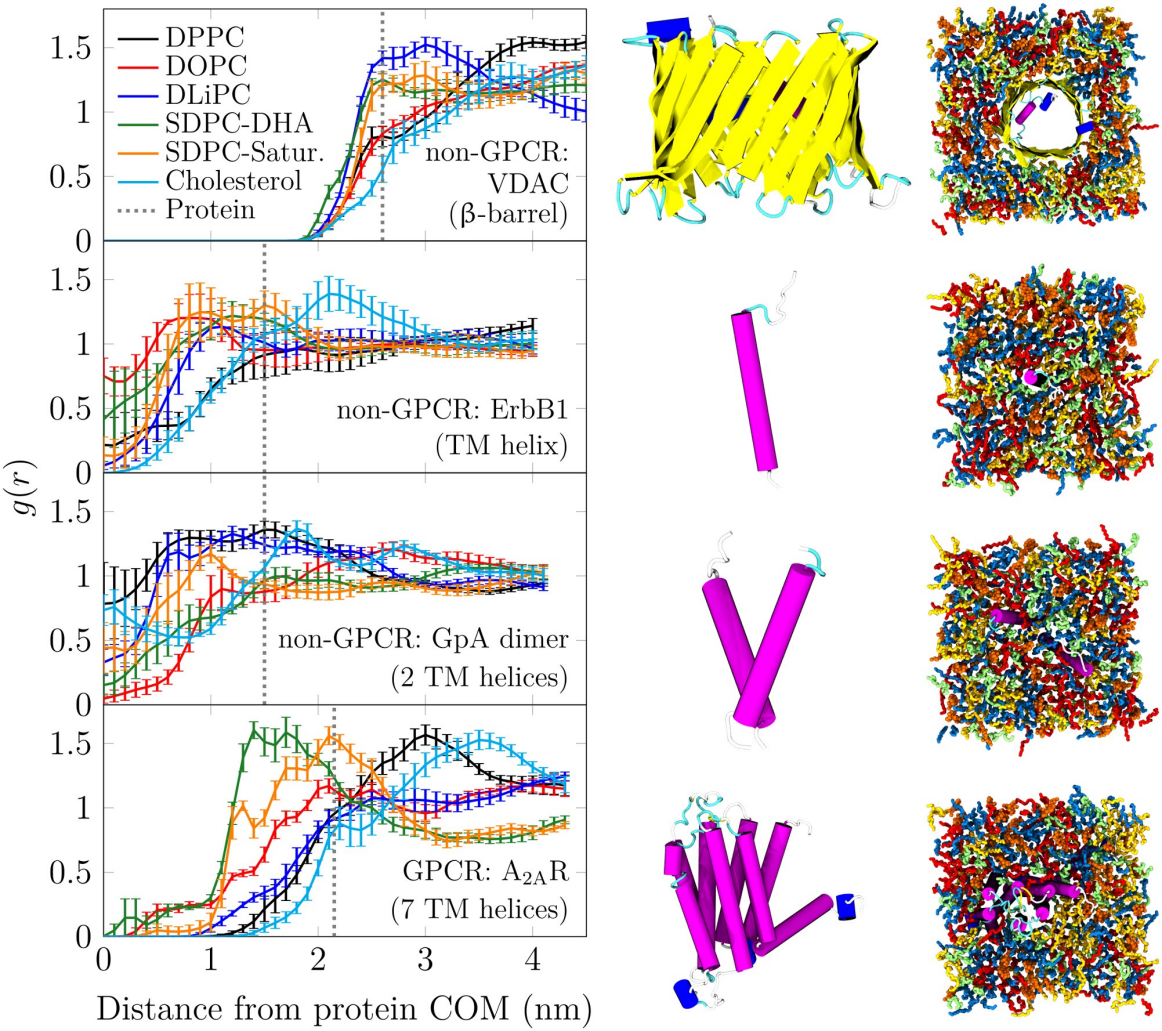
Radial distribution functions of different lipid chains around the centers of mass of four representative proteins. Leftmost column: RDF plots. The distance was measured in the membrane plane, and the last 500 ns of the simulations were used in the analysis. The gmx rdf tool was used. Error bars show standard error. The dashed line shows the approximate position of the protein surface. Two rightmost columns: Protein structures used in the solvation simulations, as well as snapshots of them residing in the membranes. For membranes, only fatty acid chains are rendered with the protein. The coloring is as follows: blue for saturated chains (both DPPC chains and the stearic acid chain of SDPC), red for monounsaturated chains (both DOPC chains), yellow for diunsaturated chains (both DLiPC chains), green for polyunsaturated chains (docosahexaenoic acid chain of SDPC), and orange for cholesterol.

Previous simulations and experiments have demonstrated the favorable interactions of DHA and GPCRs, including A_2A_R and dopamine D_2_ receptor (D_2_R) [18, 20–23, 43]. Here, we systematically studied four distinct membrane protein types—one *β*-barrel and three *α*-helical structures with 1, 2, or 7 transmembrane passes, including A_2A_R as a representative GPCR. The proteins are 1) the transmembrane domain of the human receptor tyrosine kinase (ErbB1, PDB id: 2M0B), a single helix; 2) a dimer formed by two Glycophorin A peptides [44] (GpA dimer, PDB id: 1AFO); 3) A_2A_R (PDB id: 3EML) [45], a heptahelical bundle employed in the CG free energy calculation; and 4) the voltage-dependent anion channel (VDAC, PDB id: 3EMN) [46], a *β*-barrel. These proteins are depicted in the middle column of Fig 5. Notably, the lengths of the hydrophobic spans of the helical proteins were all equal to 3.2 nm [40], so this factor cannot lead to differences in lipid–protein interactions. However, the *β*-barrel is substantially thinner at 2.3 nm.

We simulated these proteins in membranes comprised of lipids, whose chains’ unsaturation level was varied (chains with 0, 1, 2, or 6 double bonds per chain). We evaluated how the lipids solvated the proteins in these membranes using unbiased all-atom simulations together with the CHARMM36 force field [41, 42]. We paired all lipid chains with a PC head group in order to study only the effect of lipid chains. The final structures of the simulated systems are shown in the rightmost column of Fig 5. The details are given in Methods and in Section A.4 in the S1 File. The RDFs of the fatty acid chains around the proteins were determined after full lipid mixing had taken place.

It is evident from these RDFs (see leftmost column of Fig 5) that the non-GPCR proteins (here ErbB1, GpA dimer, and VDAC) do not show any clear preference for DHA. Meanwhile, A_2A_R, as a representative example of GPCRs, interacts mostly with the DHA chain of SDPE, and the saturated chain of SDPE again forms an outer layer of the lipid corona that is in contact with the protein. This observation, in agreement with the results of CG simulations (Fig 4A and our earlier study [23]), suggests that DHA adapts to the rough surface of A_2A_R. Protein roughness (*i.e*. the degree of irregularity of a protein surface) [47] is known to correlate with its propensity to interact with small molecules [48]. Therefore, it has been used to predict binding sites at the protein surface [49]. Importantly, surface roughness is a general feature of GPCRs [50] and explains the preferential interaction of the flexible and kinked DHA chain with A_2A_R [19]. The fact that a smoother *β*-barrel (VDAC) surface is not solvated by DHA is in favor of this view. Since this phenomenon is also not observed for proteins with a smaller number of helices (ErbB1 and GpA dimer), its origin likely lies in the preference of DHA for the creviced tertiary structure instead of the helical secondary structure.

**Fig 6:**
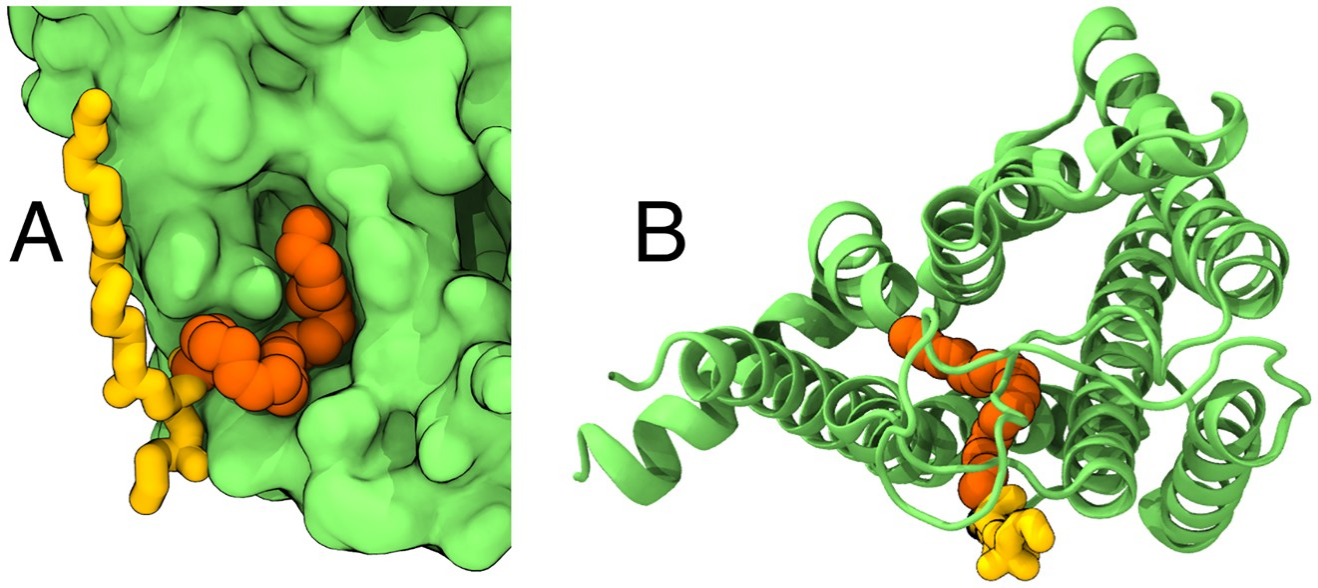
Observed conformations of SDPE due to the flexibility of DHA in the fine-grained simulations. A) An example configuration, where the DHA chain adapts to the A_2A_R surface. B) An example configuration, where the DHA chain penetrates into the core of A_2A_R. The DHA chain is shown in orange, the rest of the lipid (including the stearic acid chain) in yellow, and A_2A_R in green.

To further quantify the DHA adaptation onto the A_2A_R surface, we calculated the mean number of the residues in the helical TM region of A_2A_R that were in the vicinity (<0.3 nm) of a lipid chain in the fine-grained simulations. We found a systematic increase: +10% for the membrane with 4 mol% of SDPE and +15% for the membrane with 8 mol% of SDPE as compared to the SDPE-free case. This effect was not dependent on the chosen cutoff, as values of +7% and +17% were calculated for a cutoff of 0.4 nm. While this calculation clearly shows that DHA chains adapt better to the A_2A_R surface, a further and more systematic study on the effects of the surface topology and the amino acid content therein on DHA–protein interactions is required in the future to verify our findings. The favorable interaction between flexible DHA chains and GPCR surfaces is highlighted in Fig 6, which shows representative configurations sampled in the fine-grained all-atom simulations, where a DHA chain has adapted its conformation to the rough protein surface and entered a crevice on the A_2A_R surface (see Fig 6A), or penetrated into the helical bundle of A_2A_R (see Fig 6B).

Concluding, hybrid lipids with a DHA (or likely other PUFA) chain and a saturated chain seem to be favored by GPCRs, and this is likely due to the rough surface of the transmembrane region in these multi-helical proteins.

### SDPE-induced partitioning to L_o_ as a general feature of helical multi-pass proteins

Based on the observation that the DHA–protein interaction is characteristic for proteins with multi-pass helical bundles, we extended our free energy of transfer calculation in the CG scheme to two additional proteins of this kind. We also note that the effects for other membrane protein types might be similar in the Martini scheme as many proteins seem to interact favorably with PUFAs [51], likely due to unbalanced entropic and enthalpic contributions to this interaction. However, based on our all-atom simulations, we abstain from studying the free energies of transfer for protein types without multiple TM helices. D_2_R is linked to many neurological and psychiatric disorders [52] associated with lowered PUFA levels [10, 11, 14]. The DHA–D_2_R interaction was recently demonstrated by us [23]. We also considered the brain-associated glucose transporter GLUT1, whose function is also dependent on PUFAs [53, 54]. While GLUT1 is not a GPCR, it also has a multi-pass structure consisting of 12 helices.

We estimated the free energies of transfer for all three proteins—D_2_R, GLUT1, and A_2A_R—in the absence and in the presence (16 mol%) of SDPE and hence DHA. We also note that while the phase-separation of the commonly used lipid mixtures in the Martini model is complete and the phase boundaries are sharp [55], experiments report less distinct compositions between the L_o_ and L_d_ phases [56]. We therefore considered both the situation mimicking complete separation (such as above), as well as a more realistic situation in which the L_o_ phase had a realistic DSPC/DOPC ratio of 2.3, which is reversed during the mutation into an L_d_ phase (see Methods and Section A.2 in S1 File).

The free energies of transfer for all three proteins are shown in Fig 3B. The effect of SDPE is clearly demonstrated for all proteins. Moreover, while the absolute values are smaller in the membranes with more realistic compositions, the change of sign, *i.e*. the change in the favored phase changes consistently upon the addition of SDPE. This behavior is in line with the two phases now being less distinct, as demonstrated by the order parameters shown in Fig. D in the S1 File. Moreover, the strong association of D_2_R and GLUT1 with DHA (see Fig. F in the S1 File) is again responsible for the effect—similar to what was observed for A_2A_R (see Fig 4A). It is also worth pointing out that while we paired DHA with a PE head group (to form SDPE), the calculations performed with SDPC instead of SDPE show an almost equal effect on protein partitioning (see Section B.8 in S1 File).

Concluding, the SDPE-induced partitioning to the L_o_ phase is reproduced across three multi-helical brain-associated proteins—two of which are GPCR neuroreceptors—whose function is compromised by changes in membrane DHA levels. This effect is also consistently observed with less distinct and more realistic phase compositions.

## Discussion

Using multi-scale simulations and free energy calculations, we showed that a small amount of SDPE, a DHA-containing hybrid lipid, enhances A_2A_R partitioning to the L_o_ phase. Without DHA, the protein favors partitioning to the L_d_ phase instead. The change in this behavior stems from the rough surface of A_2A_R that favors interacting with DHA and, presumably, also with other PUFAs over saturated chains. This interaction leads to a well-organized SDPE corona where the DHA chains face the receptor, while the saturated chain of SDPE in the outer layer of the corona interacts with CHOL and saturated phospholipid chains in the L_o_ phase. Through this mechanism, the perturbations of the flexible DHA chains and the rough receptor surface on the L_o_ phase are largely eliminated. The striking finding made in this work is that the lipid corona could play a decisive role in the partitioning of membrane proteins. We showed that this holds true for A_2A_R used in this work as a prototypical GPCR. The additional results strongly suggest that the same conclusion holds for helical multi-pass proteins such as GPCRs with rough surfaces, yet not for other protein topologies with smoother surfaces.

We acknowledge that while coarse-grained models are designed to capture the correct trends, the absolute free energy values should be taken with caution. Still, our values are in line with [32] if not smaller than (compare the data for WALP23 in Methods with Ref. [55]) the values obtained with the Martini model exploiting different free energy techniques. We discuss other possible methodological limitations in detail in Section B.7 in the S1 File.

Our results suggest that small concentrations of lipids not included in model membranes might have drastic effects on the partitioning behavior of membrane proteins studied *in vivo*, and can explain why raft-associated proteins partition to the L_d_ phase in phase-separated giant unilamellar vesicles [33]. Further, the present simulation results are in line with experiments suggesting that other structural features such as post-translational modifications, protein surface roughness, and hydrophobic mismatch modulate the affinity of membrane proteins for lipid rafts [32]. Given that the solvation of a GPCR by a DHA-containing hybrid lipid is based on a layer where DHA stands next to the protein surface and saturated chains occupy the outermost shell of the protein, this arrangement can increase the raft affinity of the GPCR protein in three ways: it provides the protein with non-covalently bound saturated lipid anchors, it complements the surface roughness of the protein, and with an appropriate choice of the saturated chain in the hybrid lipids, hydrophobic mismatch can be reduced.

The concentration of DHA in raft membrane domains in the brain of healthy subjects is ∼7 mol-% [11]. Assuming a protein area coverage similar to that in red blood cells [57] and an average protein and lipid area of 10 nm^2^ and 0.7 nm^2^, respectively, the protein-to-lipid ratio would be approximately 1 to 50 per leaflet. With an SDPE content of ∼14 mol-%, and considering that the membrane has two leaflets, the estimated protein-to-SDPE ratio is 1 to 13. Strikingly, the saturation of the A_2A_R surface in our simulations takes place around this protein-to-SDPE ratio (see Figs. G and H in the S1 File). Hence, this consideration suggests that in the brain tissue of healthy subjects the DHA concentration is sufficiently large to favor the partitioning of A_2A_R to ordered regions with structural similarity to the L_o_ phase. However, one has to keep in mind that our simplified model membranes do not capture either the heterogeneity or leaflet asymmetry of membranes in the brain, which can fine-tune the partitioning behavior of proteins. Moreover, the membranes considered in this study are planar, yet GPCRs with high intrinsic curvature are also sorted by curvature [58], and the DHA corona might have an effect therein. Studies of asymmetry or curvature are beyond this work, yet might need to be taken into account when experimental validation for our findings is sought.

Then what happens if the DHA level is decreased? It is known that the level of DHA in the brain of people suffering from neurodegenerative diseases is substantially reduced [10, 11, 59]. It is tempting to speculate that the reduced DHA content would alter the partitioning of A_2A_ or D_2_ receptors, displacing them from CHOL-rich domains to disordered regions, compromising GPCR signaling. It has been shown that cholesterol binds to GPCRs such as beta-2-adrenergic receptor in an allosteric manner [6], affecting its conformational distribution, thus the concern of compromised GPCR signalling due to a lowered DHA level is justified. In brief, the effect observed in the present study on partitioning has implications on health. While DHA is promising in the treatment of neurodegenerative diseases [60], the mechanism behind this protective effect, despite rendering membranes more fluid, remains elusive. In our earlier study [23], we showed that the formation of A_2A_R homo- and hetero-oligomers with the dopamine D_2_ receptor is decreased when the DHA levels are reduced. In the current work, we postulate that DHA-containing lipids have a dual role in preventing neurodegenerative diseases by lipid–protein interactions: 1) they can influence raft partitioning, therefore indirectly 2) modulating key aspects of the GPCR biology, such as protein oligomerization. The proper function of these oligomeric and mutually regulatory receptor units in a suitable lipid environment is essential for the properly functioning healthy brain. Our findings could explain some of the beneficial effects of DHA-based therapies previously shown for certain brain disorders [61].

## Methods

All simulations are listed in Table A in the S1 File. The approach for extracting free energies of transfer is described in Section A.1 in the S1 File, and details of simulation models and methods are given in Sections A.2–A.4 of the S1 File.

### Coarse-grained simulations of protein partitioning

We embedded A_2A_R (PDB id: 3EML [45]) to an L_o_ membrane consisting of DSPC and 20 mol% CHOL. Next, varying amounts of DSPC was replaced by the hybrid lipid SDPE with a saturated (C18:0) and a polynsaturated (DHA) chain. The protein and the lipids were modeled in the coarse-grained (CG) scheme using the non-polarizable Martini 2.2 model [34–36] together with the elastic network for A_2A_R [62].

Next, DSPC was transformed into DOPC, resulting in the change of membrane phase from L_o_ to L_d_. This process was performed as an alchemical transformation using the dual topology paradigm with 27 windows. We verified the change in phase thoroughly (see Section A.1 in the S1 File), and validated our approach using the 27-residue WALP peptide that favored the L_d_ phase (free energy of transfer of 17.2±1.0 kJ/mol), in line with eperiments and simulations [55]. The associated free energy changes were estimated by the Bennett acceptance ratio (BAR) method [63] implemented in the gmx bar tool of GROMACS, and the free energy of transfer was obtained as $\Delta G^{{\text{L}}_{{\text{o}}_{\text{Prot}}}\to {\text{L}}_{{\text{d}}_{\text{Prot}}}}-\Delta G^{{\text{L}}_{\text{o}}\to {\text{L}}_{\text{d}}}$ where the two terms correspond to this phase change in the presence and absence of the protein.

To study the generality of the effect of SDPE on the partitioning of helical multi-pass membrane proteins, we considered two additional brain-associated cases, with relation to DHA—dopamine D_2_ receptor (D_2_R) and glucose transporter GLUT1 (PDB id: 4PYP [64]), whose free energies of transfer were calculated in the absence of SDPE and in the presence of 16 mol%SDPE. The systems were set up identically to the ones containing A_2A_R, and the same equilibration and simulation protocols were followed. In the simulations, performed using GROMACS v5.0.x [65], the recently suggested “New-RF” simulation parameters [66] were employed. See Section A.2 in the S1 File for further details.

Finally, the free energies of transfer were also calculated for A_2A_R, D_2_R, and GLUT1 in the absence of and in the presence of 16 mol% SDPE in membranes whose compositions mimicked those of coexisting phases in model membranes (see Table A in the S1 File).

### All-atom simulations of the effects of DHA

To study how DHA affects the adaptation of the protein into the membrane, we fine-grained the well-equilibrated CG systems containing 0, 4, and 8 mol% SDPE into all-atom resolution using the backward tool [67]. Additionally, we simulated membranes with identical lipid ratios yet in the absence of the protein as a control. All all-atom simulations, performed using GROMACS v5.0.x [65], employed the CHARMM36 force field [41, 42]. The last 150 ns of 200 ns simulations was used in the analyses. The default input parameters provided by CHARMM-GUI were used [68]. See Section A.3 in the S1 File for further details.

### All-atom simulations of the solvation of proteins by DHA

We studied whether certain protein types are more prone to be solvated by DHA in all-atom detail. To this end, we simulated four structurally different transmembrane proteins: 1) the transmembrane domain of the human receptor tyrosine kinase (ErbB1, PDB id: 2M0B), a single helix; 2) a dimer formed by two Glycophorin A peptides [44] (GpA dimer, PDB id: 1AFO); 3) A_2A_R (PDB id: 3EML) [45], a heptahelical bundle employed in the CG free energy calculation; and 4) the voltage-dependent anion channel (VDAC, PDB id: 3EMN) [46], a *β*-barrel. These proteins were embedded in a lipid bilayer consisting of equimolar concentrations of CHOL, dipalmitoyl-phosphatidylcholine (DPPC, two saturated chains; di-16:0), DOPC (two monounsaturated chains; di-18:1), dilinoleoyl-phosphatidylcholine (DLiPC, two diunsaturated chains; di-18:2), and stearoyl-docosahexaenoyl-phosphatidylcholine (SDPC, one saturated 18:0 chain and one polyunsaturated 22:6 (DHA) chain). The input structures for GROMACS were generated using the CHARMM-GUI Membrane Builder [68], and the systems were simulated for 4 μs using the input parameters provided by CHARMM-GUI [68]. The last 500 ns were used in the analyses. See Section A.4 in the S1 File for further details.

## Supporting information

S1 File A more detailed description of the methods and additional results. Thorough description of the method for estimating the free energies of transfer. Description of the setup of all simulated systems, the used simulation parameters, and the performed analyses. Additional results considering the L_o_→L_d_ transition, the PUFA–protein interactions, cholesterol binding onto the A_2A_R surface, as well as on the mechanism through which PUFAs alter the partitioning tendency of proteins.
(PDF)

## References

[1] Sezgin E, Levental I, Mayor S, Eggeling C. The Mystery of Membrane Organization: Composition, Regulation and Roles of Lipid Rafts. Nat Rev Mol Cell Biol. 2017;18(6):361–374. 10.1038/nrm.2017.16 28356571 PMC5500228

[2] Allen JA, Halverson-Tamboli RA, Rasenick MM. Lipid Raft Microdomains and Neurotransmitter Signalling. Nat Rev Neurosci. 2007;8(2):128–140. 10.1038/nrn2059 17195035

[3] Oates J, Watts A. Uncovering the Intimate Relationship between Lipids, Cholesterol and GPCR Activation. Curr Opin Struct Biol. 2011;21(6):802–807. 10.1016/j.sbi.2011.09.007 22036833

[4] Gimpl G. Interaction of G Protein Coupled Receptors and Cholesterol. Chem Phys Lipids. 2016;199:61–73. 10.1016/j.chemphyslip.2016.04.006 27108066

[5] Casiraghi M, Damian M, Lescop E, Point E, Moncoq K, Morellet N, et al Functional Modulation of a G Protein-Coupled Receptor Conformational Landscape in a Lipid Bilayer. J Am Chem Soc. 2016;138(35):11170–11175. 10.1021/jacs.6b04432 27489943

[6] Manna M, Niemelä M, Tynkkynen J, Javanainen M, Kulig W, Müller DJ, et al Mechanism of Allosteric Regulation of *β*2-Adrenergic Receptor by Cholesterol. eLife. 2016;5:e18432 10.7554/eLife.18432 27897972 PMC5182060

[7] Guixà-González R, Albasanz JL, Rodriguez-Espigares I, Pastor M, Sanz F, Martí-Solano M, et al Membrane Cholesterol Access into a G-Protein-Coupled Receptor. Nat Commun. 2017;8:14505 10.1038/ncomms14505 28220900 PMC5321766

[8] Maccarrone M, Bernardi G, Agrò aF, Centonze D. Cannabinoid Receptor Signalling in Neurodegenerative Diseases: A Potential Role for Membrane Fluidity Disturbance. Br J Pharmacol. 2011;163(7):1379–1390. 10.1111/j.1476-5381.2011.01277.x 21323908 PMC3165948

[9] Innis SM. Dietary Omega 3 Fatty Acids and the Developing Brain. Brain Res. 2008;1237:35–43. 10.1016/j.brainres.2008.08.078 18789910

[10] Martín V, Fabelo N, Santpere G, Puig B, Marín R, Ferrer I, et al Lipid Alterations in Lipid Rafts from Alzheimer’s Disease Human Brain Cortex. J Alzheimers Dis. 2010;19(2):489–502. 10.3233/JAD-2010-1242 20110596

[11] Fabelo N, Martín V, Santpere G, Marín R, Torrent L, Ferrer I, et al Severe Alterations in Lipid Composition of Frontal Cortex Lipid Rafts from Parkinson’s Disease and Incidental Parkinson’s Disease. Mol Med. 2011;17(9):1107–1118. 10.2119/molmed.2011.00119 21717034 PMC3188884

[12] Duraisamy Y, Lambert D, O’Neill CA, Padfield PJ. Differential Incorporation of Docosahexaenoic Acid into Distinct Cholesterol-Rich Membrane Raft Domains. Biochem Biophys Res Commun. 2007;360(4):885–890. 10.1016/j.bbrc.2007.06.152 17631858

[13] Konyakhina TM, Feigenson GW. Phase Diagram of a Polyunsaturated Lipid Mixture: Brain Sphingomyelin/1-Stearoyl-2-Docosahexaenoyl-Sn-Glycero-3-Phosphocholine/Cholesterol. BBA-Biomembranes. 2016;1858(1):153–161. 10.1016/j.bbamem.2015.10.016 26525664 PMC4664456

[14] Müller CP, Reichel M, Mühle C, Rhein C, Gulbins E, Kornhuber J. Brain Membrane Lipids in Major Depression and Anxiety Disorders. BBA-Mol Cell Biol L. 2015;1851(8):1052–1065. 10.1016/j.bbalip.2014.12.014 25542508

[15] Mitchell DC, Straume M, Litman BJ. Role of sn-1-Saturated,sn-2-Polyunsaturated Phospholipids in Control of Membrane Receptor Conformational Equilibrium: Effects of Cholesterol and Acyl Chain Unsaturation on the Metarhodopsin I—Metarhodopsin II Equilibrium. Biochemistry. 1992;31:662–670. 10.1021/bi00118a005 1731921

[16] Mitchell DC, Niu SL, Litman BJ. Enhancement of G Protein-Coupled Signaling by DHA Phospholipids. Lipids. 2003;38(4):437–443. 10.1007/s11745-003-1081-1 12848291

[17] Bennett MP, Mitchell DC. Regulation of Membrane Proteins by Dietary Lipids: Effects of Cholesterol and Docosahexaenoic Acid Acyl Chain-Containing Phospholipids on Rhodopsin Stability and Function. Biophys J. 2008;95(3):1206–1216. 10.1529/biophysj.107.122788 18424497 PMC2479576

[18] Feller SE, Gawrisch K, Woolf TB. Rhodopsin Exhibits a Preference for Solvation by Polyunsaturated Docosohexaenoic Acid. J Am Chem Soc. 2003;125(15):4434–4435. 10.1021/ja0345874 12683809

[19] Grossfield A, Feller SE, Pitman MC. Contribution of Omega-3 Fatty Acids to the Thermodynamics of Membrane Protein Solvation. J Phys Chem B. 2006;110(18):8907–8909. 10.1021/jp060405r 16671691

[20] Pitman MC, Grossfield A, Suits F, Feller SE. Role of Cholesterol and Polyunsaturated Chains in Lipid-Protein Interactions: Molecular Dynamics Simulation of Rhodopsin in a Realistic Membrane Environment. J Am Chem Soc. 2005;127(13):4576–4577. 10.1021/ja042715y 15796514

[21] Grossfield A, Feller SE, Pitman MC. A Role for Direct Interactions in the Modulation of Rhodopsin by *ω*-3 Polyunsaturated Lipids. Proc Natl Acad Sci USA. 2006;103(13):4888–4893. 10.1073/pnas.0508352103 16547139 PMC1458765

[22] Horn JN, Kao TC, Grossfield A. Coarse-Grained Molecular Dynamics Provides Insight into the Interactions of Lipids and Cholesterol with Rhodopsin In: G Protein-Coupled Receptors—Modeling and Simulation. Springer; 2014 p. 75–94. 10.1007/978-94-007-7423-0_5 PMC4034522 24158802

[23] Guixà-González R, Javanainen M, Gómez-Soler M, Cordobilla B, Domingo JC, Sanz F, et al Membrane Omega-3 Fatty Acids Modulate the Oligomerisation Kinetics of Adenosine A_2A_ and Dopamine D_2_ Receptors. Sci Rep. 2016;6:19839 10.1038/srep19839 26796668 PMC4726318

[24] Chen JF, Eltzschig HK, Fredholm BB. Adenosine Receptors as Drug Targets–What Are the Challenges? Nat Rev Drug Discov. 2013;12(4):265–286. 10.1038/nrd3955 23535933 PMC3930074

[25] Jorg M, Scammells P, Capuano B. The Dopamine D_2_ and Adenosine A_2A_ Receptors: Past, Present and Future Trends for the Treatment of Parkinson’s Disease. Curr Med Chem. 2014;21(27):3188–3210. 10.2174/1389200215666140217110716 24533801

[26] Liu W, Chun E, Thompson AA, Chubukov P, Xu F, Katritch V, et al Structural Basis for Allosteric Regulation of GPCRs by Sodium Ions. Science. 2012;337(6091):232–236. 10.1126/science.1219218 22798613 PMC3399762

[27] Lee JY, Lyman E. Predictions for Cholesterol Interaction Sites on the A_2A_ Adenosine Receptor. J Am Chem Soc. 2012;134(40):16512–16515. 10.1021/ja307532d 23005256 PMC3652312

[28] Rouviere E, Arnarez C, Yang L, Lyman E. Identification of Two New Cholesterol Interaction Sites on the A_2A_ Adenosine Receptor. Biophys J. 2017;113(11):2415–2424. 10.1016/j.bpj.2017.09.027 29211995 PMC5738547

[29] Lam RS, Nahirney D, Duszyk M. Cholesterol-Dependent Regulation of Adenosine A_2A_ Receptor-Mediated Anion Secretion in Colon Epithelial Cells. Exp Cell Res. 2009;315(17):3028–3035. 10.1016/j.yexcr.2009.06.005 19523941

[30] Thurner P, Gsandtner I, Kudlacek O, Choquet D, Nanoff C, Freissmuth M, et al A Two-State Model for the Diffusion of the A_2A_ Adenosine Receptor in Hippocampal Neurons: Agonist-Induced Switch to Slow Mobility is Modified by Synapse-Associated Protein 102 (SAP102). J Biol Chem. 2014;289(13):9263–9274. 10.1074/jbc.M113.505685 24509856 PMC3979375

[31] Lasley RD. Adenosine Receptors and Membrane Microdomains. BBA-Biomembranes. 2011;1808(5):1284–1289. 10.1016/j.bbamem.2010.09.019 20888790 PMC3042032

[32] Lorent JH, Diaz-Rohrer B, Lin X, Spring K, Gorfe AA, Levental KR, et al Structural Determinants and Functional Consequences of Protein Affinity for Membrane Rafts. Nat Commun. 2017;8(1):1219 10.1038/s41467-017-01328-3 29089556 PMC5663905

[33] Schlebach JP, Barrett PJ, Day CA, Kim JH, Kenworthy AK, Sanders CR. Topologically Diverse Human Membrane Proteins Partition to Liquid-Disordered Domains in Phase-Separated Lipid Vesicles. Biochemistry. 2016;55(7):985–988. 10.1021/acs.biochem.5b01154 26859249 PMC4766968

[34] Marrink SJ, Risselada HJ, Yefimov S, Tieleman DP, De Vries AH. The MARTINI Force Field: Coarse Grained Model for Biomolecular Simulations. J Phys Chem B. 2007;111(27):7812–7824. 10.1021/jp071097f 17569554

[35] Monticelli L, Kandasamy SK, Periole X, Larson RG, Tieleman DP, Marrink SJ. The MARTINI Coarse-grained Force Field: Extension to Proteins. J Chem Theory Comput. 2008;4(5):819–834. 10.1021/ct700324x 26621095

[36] de Jong DH, Singh G, Bennett WD, Arnarez C, Wassenaar TA, Schäfer LV, et al Improved Parameters for the Martini Coarse-Grained Protein Force Field. J Chem Theory Comput. 2012;9(1):687–697. 10.1021/ct300646g 26589065

[37] Langelier B, Linard A, Bordat C, Lavialle M, Heberden C. Long Chain-polyunsaturated Fatty Acids Modulate Membrane Phospholipid Composition and Protein Localization in Lipid Rafts of Neural Stem Cell Cultures. J Cell Biochem. 2010;110(6):1356–1364. 10.1002/jcb.22652 20564231

[38] Zhao J, Wu J, Heberle FA, Mills TT, Klawitter P, Huang G, et al Phase Studies of Model Biomembranes: Complex Behavior of DSPC/DOPC/cholesterol. BBA-Biomembranes. 2007;1768(11):2764–2776. 10.1016/j.bbamem.2007.07.008 17825247 PMC2701629

[39] Mouritsen O, Bloom M. Mattress Model of Lipid–Protein Interactions in Membranes. Biophys J. 1984;46(2):141–153. 10.1016/S0006-3495(84)84007-2 6478029 PMC1435039

[40] Lomize MA, Pogozheva ID, Joo H, Mosberg HI, Lomize AL. OPM Database and PPM Web Server: Resources for Positioning of Proteins in Membranes. Nucleic Acids Res. 2011;40(D1):D370–D376. 10.1093/nar/gkr703 21890895 PMC3245162

[41] Klauda JB, Venable RM, Freites JA, O’Connor JW, Tobias DJ, Mondragon-Ramirez C, et al Update of the CHARMM All-Atom Additive Force Field for Lipids: Validation on Six Lipid Types. J Phys Chem B. 2010;114(23):7830–7843. 10.1021/jp101759q 20496934 PMC2922408

[42] Best RB, Zhu X, Shim J, Lopes PE, Mittal J, Feig M, et al Optimization of the Additive CHARMM All-Atom Protein Force Field Targeting Improved Sampling of the Backbone *ϕ*, *ψ* and Side-Chain *χ*1 and *χ*2 Dihedral Angles. J Chem Theory Comput. 2012;8(9):3257–3273. 10.1021/ct300400x 23341755 PMC3549273

[43] Soubias O, Teague WE, Gawrisch K. Evidence for Specificity in Lipid–Rhodopsin Interactions. J Biol Chem. 2006;281(44):33233–33241. 10.1074/jbc.M603059200 16959786

[44] MacKenzie KR, Prestegard JH, Engelman DM. A Transmembrane Helix Dimer: Structure and Implications. Science. 1997;276(5309):131–133. 10.1126/science.276.5309.131 9082985

[45] Jaakola VP, Griffith MT, Hanson MA, Cherezov V, Chien EY, Lane JR, et al The 2.6 Angstrom Crystal Structure of a Human A_2A_ Adenosine Receptor Bound to an Antagonist. Science. 2008;322(5905):1211–1217. 10.1126/science.1164772 18832607 PMC2586971

[46] Ujwal R, Cascio D, Colletier JP, Faham S, Zhang J, Toro L, et al The Crystal Structure of Mouse VDAC1 at 2.3 Å Resolution Reveals Mechanistic Insights into Metabolite Gating. Proc Natl Acad Sci USA. 2008;105(46):17742–17747. 10.1073/pnas.0809634105 18988731 PMC2584669

[47] Lewis M, Rees DC. Fractal Surfaces of Proteins. Science. 1985;230(4730):1163–1165. 10.1126/science.4071040 4071040

[48] Pettit FK, Bowie JU. Protein Surface Roughness and Small Molecular Binding Sites. J Mol Biol. 1999;285(4):1377–1382. 10.1006/jmbi.1998.2411 9917382

[49] Banerji A, Navare C. Fractal Nature of Protein Surface Roughness: a Note on Quantification of Change of Surface Roughness in Active Sites, Before and After Binding. J Mol Recognit. 2013;26(5):201–214. 10.1002/jmr.2264 23526774

[50] Kaczor AA, Guixà-González R, Carrió P, Obiol-Pardo C, Pastor M, Selent J. Fractal Dimension as a Measure of Surface Roughness of G Protein-Coupled Receptors: Implications for Structure and Function. J Mol Model. 2012;18(9):4465–4475. 10.1007/s00894-012-1431-2 22643967 PMC3429779

[51] Corradi V, Mendez-Villuendas E, Ingólfsson HI, Gu RX, Siuda I, Melo MN, et al Lipid–Protein Interactions are Unique Fingerprints for Membrane Proteins. ACS Cent Sci. 2018;4(6):709–717. 10.1021/acscentsci.8b00143 29974066 PMC6028153

[52] Beaulieu JM, Gainetdinov RR. The Physiology, Signaling, and Pharmacology of Dopamine Receptors. Pharmacol Rev. 2011;63(1):182–217. 10.1124/pr.110.002642 21303898

[53] Ximenes da Silva A, Lavialle F, Gendrot G, Guesnet P, Alessandri JM, Lavialle M. Glucose Transport and Utilization Are Altered in the Brain of Rats Deficient in N-3 Polyunsaturated Fatty Acids. J Neurochem. 2002;81(6):1328–1337. 10.1046/j.1471-4159.2002.00932.x 12068080

[54] Pifferi F, Jouin M, Alessandri J, Haedke U, Roux F, Perriere N, et al N-3 Fatty Acids Modulate Brain Glucose Transport in Endothelial Cells of the Blood–Brain Barrier. Prostaglandins Leukot Essent Fatty Acids. 2007;77(5-6):279–286. 10.1016/j.plefa.2007.10.011 18042368

[55] Schäfer LV, de Jong DH, Holt A, Rzepiela AJ, de Vries AH, Poolman B, et al Lipid Packing Drives the Segregation of Transmembrane Helices into Disordered Lipid Domains in Model Membranes. Proc Natl Acad Sci USA. 2011;108(4):1343–1348. 10.1073/pnas.1009362108 21205902 PMC3029762

[56] Uppamoochikkal P, Tristram-Nagle S, Nagle JF. Orientation of Tie-Lines in the Phase Diagram of Dopc/Dppc/Cholesterol Model Biomembranes. Langmuir. 2010;26(22):17363–17368. 10.1021/la103024f 20968281 PMC2978278

[57] Dupuy AD, Engelman DM. Protein Area Occupancy at the Center of the Red Blood Cell Membrane. Proc Natl Acad Sci USA. 2008;105(8):2848–2852. 10.1073/pnas.0712379105 18287056 PMC2268548

[58] Rosholm KR, Leijnse N, Mantsiou A, Tkach V, Pedersen SL, Wirth VF, et al Membrane Curvature Regulates Ligand-specific Membrane Sorting of GPCRs in Living Cells. Nat Chem Biol. 2017;13(7):724 10.1038/nchembio.2372 28481347

[59] du Bois TM, Deng C, Huang XF. Membrane Phospholipid Composition, Alterations in Neurotransmitter Systems and Schizophrenia. Prog Neuropsychopharmacol Biol Psychiatry. 2005;29(6):878–888. 10.1016/j.pnpbp.2005.04.034 16005134

[60] Bousquet M, Calon F, Cicchetti F. Impact of Omega-3 Fatty Acids in Parkinson’s Disease. Ageing Res Rev. 2011;10(4):453–463. 10.1016/j.arr.2011.03.001 21414422

[61] Bazinet RP, Layé S. Polyunsaturated Fatty Acids and Their Metabolites in Brain Function and Disease. Nat Rev Neurosci. 2014;15(12):771–785. 10.1038/nrn3820 25387473

[62] Periole X, Cavalli M, Marrink SJ, Ceruso MA. Combining an Elastic Network with a Coarse-Grained Molecular Force Field: Structure, Dynamics, and Intermolecular Recognition. J Chem Theory Comput. 2009;5(9):2531–2543. 10.1021/ct9002114 26616630

[63] Bennett CH. Efficient Estimation of Free Energy Differences from Monte Carlo Data. J Comput Phys. 1976;22(2):245–268. 10.1016/0021-9991(76)90078-4

[64] Deng D, Xu C, Sun P, Wu J, Yan C, Hu M, et al Crystal Structure of the Human Glucose Transporter GLUT1. Nature. 2014;510(7503):121–125. 10.1038/nature13306 24847886

[65] Abraham MJ, Murtola T, Schulz R, Páll S, Smith JC, Hess B, et al GROMACS: High Performance Molecular Simulations through Multi-Level Parallelism from Laptops to Supercomputers. SoftwareX. 2015;1:19–25. 10.1016/j.softx.2015.06.001

[66] de Jong DH, Baoukina S, Ingólfsson HI, Marrink SJ. Martini Straight: Boosting Performance Using a Shorter Cutoff and GPUs. Comput Phys Comm. 2016;199:1–7. 10.1016/j.cpc.2015.09.014

[67] Wassenaar TA, Pluhackova K, Böckmann RA, Marrink SJ, Tieleman DP. Going Backward: A Flexible Geometric Approach to Reverse Transformation from Coarse Grained to Atomistic Models. J Chem Theory Comput. 2014;10(2):676–690. 10.1021/ct400617g 26580045

[68] Lee J, Cheng X, Swails JM, Yeom MS, Eastman PK, Lemkul JA, et al CHARMM-GUI Input Generator for NAMD, GROMACS, AMBER, OpenMM, and CHARMM/OpenMM Simulations Using the CHARMM36 Additive Force Field. J Chem Theory Comput. 2015;12(1):405–413. 10.1021/acs.jctc.5b00935 26631602 PMC4712441

