# Supplementary material for: Reduced level of docosahexaenoic acid shifts GPCR neuroreceptors to less ordered membrane regions
Source: PLoS Comput Biol. 2019 May 20;15(5):e1007033. doi: 10.1371/journal.pcbi.1007033 (PMC6544328; doi:10.1371/journal.pcbi.1007033)
Supplement: S1 File — Thorough description of the method for estimating the free energies of transfer. Description of the setup of all simulated systems, the used simulation parameters, and the performed analyses. Additional results considering the Lo→Ld transition, the PUFA–protein interactions, cholesterol binding onto the A2AR surface, as well as on the mechanism through which PUFAs alter the partitioning tendency of proteins. (PDF) [file pcbi.1007033.s001.pdf]

Supporting Information: Reduced Level of Docosahexaenoic Acid Shifts GPCR Neuroreceptors to Less Ordered Membrane Regions

Matti Javanainen[1,2,3,*], Giray Enkavi[1,2], Ramon Guixà-Gonzaléz[4], Waldemar Kulig[1,2], Hector Martinez-Seara[3], Ilya Levental[5], Ilpo Vattulainen[1,2,6,*]

1 Computational Physics Laboratory, Tampere University, POB 692, FI-33014 Tampere, Finland
2 Department of Physics, University of Helsinki, FI-00014 Helsinki, Finland
3 Institute of Organic Chemistry and Biochemistry of the Czech Academy of Sciences, CZ-16610 Prague, Czech Republic
4 Laboratory of Computational Medicine, Biostatistics Unit, Faculty of Medicine, Autonomous University of Barcelona, 08193, Bellaterra, Spain
5 Department of Integrated Biology and Pharmacology, McGovern Medical School, University of Texas Health Science Center at Houston, 77030 Houston, USA
6 MEMPHYS – Center for Biomembrane Physics

* matti.javanainen@uochb.cas.cz, ilpo.vattulainen@helsinki.fi

Contents

A Methods and Simulation Models **S2**
A.1 Estimation of the Partition Free Energy S2
A.2 Coarse-Grained Simulations of Protein Partitioning S2
A.3 All-Atom Simulations of the Effects of DHA S5
A.4 All-Atom Simulations of the Solvation of Proteins by DHA S6

B Supplementary Results **S7**
B.1 The L_o–L_d Transition is Smooth S7
B.2 Saturation of the $A_{2A}R$ Surface by DHA S10
B.3 Spatially Resolved Effects of SDPE and $A_{2A}R$ on Membrane Order . . . S14
B.4 The Presence of SDPE does not Affect Hydrophobic Mismatch S16
B.5 SDPE Shell Does Not Increase the Conformation Entropy of the Receptor S17
B.6 Convergence of the All-Atom Solvation Simulations S18
B.7 Methodological Limitations S19
B.8 Effect of DHA-containing Lipid Head Group on Partitioning S20

A Methods and Simulation Models

A.1 Estimation of the Partition Free Energy

The partition coefficient of a protein between the $\mathrm{L_d}$ and $\mathrm{L_o}$ phases is defined as

$$P_{\mathrm{L_d/L_o}} = \frac{[\mathrm{Prot}_{\mathrm{L_d}}]}{[\mathrm{Prot}_{\mathrm{L_o}}]} = \frac{n_{\mathrm{Prot}_{\mathrm{L_d}}}/V_{\mathrm{L_d}}}{n_{\mathrm{Prot}_{\mathrm{L_o}}}/V_{\mathrm{L_o}}} = \frac{x_{\mathrm{Prot}_{\mathrm{L_d}}}/V_{\mathrm{L_d}}}{x_{\mathrm{Prot}_{\mathrm{L_o}}}/V_{\mathrm{L_o}}}, \tag{1}$$

where $[\mathrm{Prot}_\phi]$ refers to the equilibrium molar concentration of the protein in the liquid-disordered ($\phi = \mathrm{L_d}$) or liquid-ordered ($\phi = \mathrm{L_o}$) phase, which by definition matches the number of proteins in the given phase (n_{Prot_ϕ}) divided by the volume of the phase (V_ϕ). Expressing molar concentrations in terms of molar fractions x_ϕ and phase volumes V_ϕ, taking the logarithm on both sides, and multiplying them with the thermal energy $k_\mathrm{B}T$, one obtains

$$-k_\mathrm{B}T \ln P_{\mathrm{L_d/L_o}} = -k_\mathrm{B}T \ln x_{\mathrm{Prot}_{\mathrm{L_d}}} + k_\mathrm{B}T \ln x_{\mathrm{Prot}_{\mathrm{L_o}}} - k_\mathrm{B}T \ln \frac{V_{\mathrm{L_o}}}{V_{\mathrm{L_d}}}. \tag{2}$$

Note that the last term in Eq. (2) vanishes when the volumes of the phases are identical, *i.e.* $V_{\mathrm{L_o}}/V_{\mathrm{L_d}} = 1$. In our study, this holds true to a very good approximation. The equation can then be simply written as the difference between the transfer free energies of the protein from vacuum to the $\mathrm{L_d}$ phase ($\Delta G^{\mathrm{Prot_{vac}} \to \mathrm{Prot}_{\mathrm{L_d}}}$) and from vacuum to the $\mathrm{L_o}$ phase ($\Delta G^{\mathrm{Prot_{vac}} \to \mathrm{Prot}_{\mathrm{L_o}}}$), thus yielding

$$-k_\mathrm{B}T \ln P_{\mathrm{L_d/L_o}} = \Delta G^{\mathrm{Prot_{vac}} \to \mathrm{Prot}_{\mathrm{L_d}}} - \Delta G^{\mathrm{Prot_{vac}} \to \mathrm{Prot}_{\mathrm{L_o}}}. \tag{3}$$

This equation can be expressed in a more convenient form by realising that there is a set of transformations inducing only a minor perturbation to the system. The thermodynamic cycle depicted in Fig. 1F in the main text shows that

$$\begin{aligned} -k_\mathrm{B}T \ln P_{\mathrm{L_d/L_o}} &= \Delta G^{\mathrm{Prot_{vac}} \to \mathrm{Prot}_{\mathrm{L_d}}} - \Delta G^{\mathrm{Prot_{vac}} \to \mathrm{Prot}_{\mathrm{L_o}}} \\ &= \Delta G^{\mathrm{L_{oProt}} \to \mathrm{L_{dProt}}} - \Delta G^{\mathrm{L_o} \to \mathrm{L_d}}, \end{aligned} \tag{4}$$

where $\mathrm{L_{oProt}} \to \mathrm{L_{dProt}}$ and $\mathrm{L_o} \to \mathrm{L_d}$ represent the transformations of the membrane from the $\mathrm{L_o}$ to the $\mathrm{L_d}$ phase in the presence and in the absence of the protein, respectively. Unlike Eq. (3), Eq. (4) can be easily implemented in MD simulations using a dual topology approach for the lipids, in which $\mathrm{L_d}$ forming lipids are changed into $\mathrm{L_o}$ forming lipids in two different sets of free energy perturbation simulations, one in the presence and another in the absence of the protein. Since the approach given on the last line of Eq. (4) induces only a small perturbation to the system, we use it as the basis of our calculations. We refer to this difference from now on as the free energy of transfer. Notably, a negative value refers to favorable partitioning to the $\mathrm{L_d}$ phase, while preferable partitioning to the $\mathrm{L_o}$ phase is characterized by a positive value.

We also validated our approach for calculating the free energies of transfer by considering a transmembrane peptide of the WALP class, which is known to partition to the $\mathrm{L_d}$ phase in both simulations and experiments [1]. In line with these findings, we obtained a free energy of transfer of 17.2±1.0 kJ/mol towards the $\mathrm{L_d}$ phase for the 27-residue WALP peptide.

A.2 Coarse-Grained Simulations of Protein Partitioning

$\mathrm{A_{2A}R}$ in its inactive state (PDB id: 3EML [2]) was embedded in an $\mathrm{L_o}$ membrane consisting of 400 distearoyl-phosphatidylcholine (DSPC) and 100 (20 mol%) CHOL molecules. To complete the cycle in Fig. 1F in the main text (see also Eq. (4)), a

Table A. A list of the performed simulations, totaling ∼0.61 ms (∼2.4 ms if reported using the common Martini speed up factor of 4) and 17.2 µs of all-atom simulation. Cholesterol is denoted as "CHOL", and "EQ" refers to equilibration time not considered in the analyses. "W" stands for windows in the free energy calculation. In Set 1, DSPC is mutated into DOPC in the free energy calculation. In systems marked with *, only part of DSPC is mutated into DOPC in a way that their initial ratio in the L_o phase is reversed in the L_d phase. For details, see the Methods section in the main paper and the rest of this section.

Composition (Lipid mol-%)	Simulation Time
**(Set 1) Free Energy of Transfer Calculations (Coarse-grained)**	
DSPC/CHOL (80/20)	200 ns (EQ) + 27 W × (50 ns (EQ) + 450 ns)
DSPC/SDPE/CHOL (76/4/20)	200 ns (EQ) + 27 W × (50 ns (EQ) + 450 ns)
DSPC/SDPE/CHOL (72/8/20)	200 ns (EQ) + 27 W × (50 ns (EQ) + 450 ns)
DSPC/SDPE/CHOL (68/12/20)	200 ns (EQ) + 27 W × (50 ns (EQ) + 450 ns)
DSPC/SDPE/CHOL (64/16/20)	200 ns (EQ) + 27 W × (50 ns (EQ) + 450 ns)
DSPC/DOPC/CHOL* (56/24/20)	27 W × (50 ns (EQ) + 450 ns)
DSPC/DOPC/SDPE/CHOL* (45/19/16/20)	27 W × (50 ns (EQ) + 450 ns)
DSPC/DOPC/SDPC/CHOL* (45/19/16/20)	27 W × (50 ns (EQ) + 450 ns)
$A_{2A}R$ + DSPC/CHOL (80/20)	15 ns (EQ) + 27 W × (100 ns (EQ) + 900 ns)
$A_{2A}R$ + DSPC/SDPE/CHOL (76/4/20)	10 µs (EQ) + 27 W × (100 ns (EQ) + 900 ns)
$A_{2A}R$ + DSPC/SDPE/CHOL (72/8/20)	10 µs (EQ) + 27 W × (100 ns (EQ) + 900 ns)
$A_{2A}R$ + DSPC/SDPE/CHOL (68/12/20)	10 µs (EQ) + 27 W × (100 ns (EQ) + 900 ns)
$A_{2A}R$ + DSPC/SDPE/CHOL (64/16/20)	10 µs (EQ) + 27 W × (100 ns (EQ) + 900 ns)
$A_{2A}R$ + DSPC/DOPC/CHOL* (56/24/20)	1 µs (EQ) + 27 W × (100 ns (EQ) + 900 ns)
$A_{2A}R$ + DSPC/DOPC/SDPE/CHOL* (45/19/16/20)	1 µs (EQ) + 27 W × (100 ns (EQ) + 900 ns)
$A_{2A}R$ + DSPC/DOPC/SDPC/CHOL* (45/19/16/20)	1 µs (EQ) + 27 W × (100 ns (EQ) + 900 ns)
D_2R + DSPC/CHOL (80/20)	15 ns (EQ) + 27 W × (100 ns (EQ) + 900 ns)
D_2R + DSPC/SDPE/CHOL (64/16/20)	10 µs (EQ) + 27 W × (100 ns (EQ) + 900 ns)
D_2R + DSPC/DOPC/CHOL* (56/24/20)	1 µs (EQ) + 27 W × (100 ns (EQ) + 900 ns)
D_2R + DSPC/DOPC/SDPE/CHOL* (45/19/16/20)	1 µs (EQ) + 27 W × (100 ns (EQ) + 900 ns)
GLUT1 + DSPC/CHOL (80/20)	15 ns (EQ) + 27 W × (100 ns (EQ) + 900 ns)
GLUT1 + DSPC/SDPE/CHOL (64/16/20)	10 µs (EQ) + 27 W × (100 ns (EQ) + 900 ns)
GLUT1 + DSPC/DOPC/CHOL* (56/24/20)	1 µs (EQ) + 27 W × (100 ns (EQ) + 900 ns)
GLUT1 + DSPC/DOPC/SDPE/CHOL* (45/19/16/20)	1 µs (EQ) + 27 W × (100 ns (EQ) + 900 ns)
**(Set 2) Effect of SDPE on Membrane (Fine-grained All-atom)**	
DSPC/CHOL (80/20)	50 ns (EQ) + 150 ns
DSPC/SDPE/CHOL (76/4/20)	50 ns (EQ) + 150 ns
DSPC/SDPE/CHOL (72/8/20)	50 ns (EQ) + 150 ns
$A_{2A}R$ + DSPC/CHOL (80/20)	50 ns (EQ) + 150 ns
$A_{2A}R$ + DSPC/SDPE/CHOL (76/4/20)	50 ns (EQ) + 150 ns
$A_{2A}R$ + DSPC/SDPE/CHOL (72/8/20)	50 ns (EQ) + 150 ns
**(Set 3) Solvatiom of Different Protein Types by Fatty Acids (All-atom)**	
$A_{2A}R$ + DPPC/DOPC/DLiPC/SDPC/CHOL	3.5 µs (EQ) + 500 ns
GpA dimer + –, , –	3.5 µs (EQ) + 500 ns
ErbB1+ –, , –	3.5 µs (EQ) + 500 ns
VDAC + –, , –	3.5 µs (EQ) + 500 ns

protein-free system with the same lipid composition was also considered. Both systems were solvated by 6250 water beads, corresponding to 50 water molecules per lipid.

sodium and 40 chloride ions were added to mimic a physiological salt concentration, and an additional set of 10 chloride counter ions was included to neutralize protein-containing systems. Next, varying numbers of DSPC were replaced by a hybrid lipid modeled as stearoyl-docosahexaenoyl-phosphatidyl-ethanolamine (SDPE), which contains both a saturated (C18:0) and a polynsaturated (DHA) chain. Since DHA preferentially incorporates into PE-containing phospholipids [3], the PE head group was chosen to host DHA. In this manner, we constructed membranes with 0, 4, 8, 12, and 16 mol% of SDPE, keeping the molar fraction of CHOL constant (20 mol%), the rest being DSPC (see Table A). The protein and the lipids were modeled in the coarse-grained (CG) scheme using the Martini force field [4–6] together with the elastic network for $A_{2A}R$ [7] (see SI for details).

Next, all the systems were equilibrated. The systems with SDPE and $A_{2A}R$ were simulated for 10 µs each to allow for lateral rearrangement of membrane lipids around the receptor. Then, in all CG systems (Table A), DSPC with a high T_m was transformed into dioleoylphosphatidylcholine (DOPC) with a low main transition temperature (T_m), in order to take the membrane from the L_o phase to the L_d phase. Although the coarse-grained lipid model does not reproduce quantitatively the experimentally determined temperature-dependent phase behavior of the studied membranes [8, 9], coarse-graining captures the qualitative differences and allows us to perform the $L_o \rightarrow L_d$ transformations at a fixed temperature and particle number. This process was performed as an alchemical transformation using the dual topology paradigm with 27 windows. In the coarse-grained scheme, the alchemical change from DSPC to DOPC corresponds to changing the chain bead types from single-bonded (C1) to double-bonded ones (C3), and modifying the corresponding angle parameters. The angles between beads describing saturated chains in the DSPC molecule (C1–C1–C1) have an equilibrium value of 180° and a force constant of 25 $kJ\,mol^{-1}\,nm^{-2}$, while for the unsaturated chain in DOPC (C1–C3–C1) the corresponding values are 120° and 45 $kJ\,mol^{-1}\,nm^{-2}$.

The protein-containing and protein-free windows of the alchemical transformation were simulated for 1 µs and 500 ns with time steps of 10 fs and 20 fs, respectively, and the first 10% of simulation was omitted in all analyses. The values of λ were 0.00, 0.015, 0.035, 0.05, 0.08, 0.11, 0.15, 0.20, 0.25, 0.30, 0.35, 0.40, 0.45, 0.50, 0.55, 0.60, 0.65, 0.70, 0.75, 0.80, 0.85, 0.89, 0.92, 0.95, 0.965, 0.985, and 1.00. The free energy differences (Eq. (4)) were estimated by the Bennett acceptance ratio (BAR) method [10] implemented in the `gmx bar` tool of GROMACS.

To study the generality of the effect of SDPE on the partitioning of helical multi-pass membrane proteins, we considered two additional cases whose functions have been suggested to be DHA-dependent. The GPCR dopamine D_2 receptor (D_2R) is linked to many neurological and psychiatric disorders [11] that are further associated with lowered PUFA levels [12–15]. The D_2R structure was adapted from our recent study [16], which demonstrated the DHA–D_2R interaction [16]. We also considered the brain-associated glucose transporter GLUT1 (PDB id: 4PYP [17]), whose function is also dependent on PUFAs [18–20]. While GLUT1 is not a GPCR, it also has a multi-pass structure consisting of 12 helices. The free energies of transfer for D_2R and GLUT1 were calculated in the absence of SDPE and in the presence of 16 mol% SDPE. The systems were set up identically to the ones containing $A_{2A}R$, and the same equilibration and simulation protocols were followed.

Finally, the calculation for these systems was repeated with somewhat less distinct phases, in agreement with experiments on model membranes [21]. To this end, part of DSPC was replaced by DOPC in the L_o phase. Only part of DSPC was then mutated into DOPC, so that the L_d phase contained the same amount of DSPC as the L_o phase contained DOPC. The DSPC/DOPC ratios were identical despite the presence or

absence of SDPE. The well-equilibrated initial conformations were taken for systems with proteins, and after the lipid replacement a further 1 µs equilibration was perfomed. Finally, the 27 windows were each simulated for 500 ns and 1 µs for protein-free and protein-containing systems, respectively.

In the simulations, the recently suggested "New-RF" simulation parameters [22] were employed. Namely, the reaction field method with a cut-off of 1.1 nm was used for electrostatics. Lennard-Jones interactions were cut off at 1.1 nm. Buffered Verlet lists [23] were employed, and the potential shift modifier was applied to non-bonded interactions. Temperature was maintained at 315 K using the stochastic velocity rescaling algorithm [24] with a time constant of 1 ps. Protein, lipids and solvent were coupled separately. Pressure was coupled semi-isotropically to the Parrinello–Rahman barostat [25] using a time constant of 12 ps and a reference pressure of 1 bar. Notably, the temperature of 315 K was chosen as the mixtures of DSPC/cholesterol and DOPC/cholesterol form L_o and L_d phase at this temperature, respectively, in the coarse-grained simulations. The leap frog integrator with a time step of 10 fs (simulations with proteins) or 20 fs (simulations without proteins) was employed. GROMACS v.5.0.x [26] was employed for all simulations.

A.3 All-Atom Simulations of the Effects of DHA

To study how DHA affects the adaptation of the protein into the membrane, we fine-grained the CG systems containing 0, 4, and 8 mol% SDPE into the all-atom resolution using the `backward` tool [27] of Martini. To this end, we used equilibrated membranes taken from CG simulations with a well-equilibrated lateral lipid distribution (after 10 µs in SDPE-containing systems and 15 ns in the SDPE-free system). After fine-graining, the number of water molecules was reduced from 25000 (6250 beads) to 20000 (40 molecules per lipid), and ∼150 mM of NaCl together with counter ions were re-added to the systems. Thus, the systems composed of 400, 380, or 360 DSPC with 0, 20, or 40 SDPE and 100 cholesterol molecules together with the $A_{2A}R$. Notably, during the fine-graining process, we used improper dihedral potential in the SDPE double bonds to keep them in the *cis* conformation. After fine-graining, the systems were energy-minimized, equilibrated, and finally simulated for 200 ns out of which the first 50 ns was omitted in the analyses. The simulations were run at 343 K and 1 bar to maintain the fluid phase (DSPC has a transition temperature of 328 K [28]). Importantly, no unfolding of the proteins was observed despite the high temperature, likely due to the fairly short simulation times.

Notably, DSPC is used as the transformation DSPC→DOPC can be performed easily in the coarse-grained scheme as the two lipids have the same number of beads. With the most recent Martini lipid model where oleic acid chains consist of four beads instead of the earlier five, a transformation of DPPC→DOPC could be used as well. However, this modification to the oleic acid chain was made after the initiation of our study.

Finally, we simulated protein-free systems with 0, 4, and 8 mol% SDPE. These membranes consisted of a total of 200 phospholipids and 50 cholesterols. They were set up using the CHARMM-GUI membrane builder [29] and hydrated with 40 water molecules per lipid and with ∼150 mM of NaCl. These membranes were simulated at 343 K and 1 bar for 200 ns out of which the first 50 ns were discarded.

The force field employed in all all-atom simulations was CHARMM36 [30, 31], and each of the systems was simulated for 200 ns using the default input parameters provided by CHARMM-GUI [32, 33]. Namely, the Verlet cutoff scheme [23] was employed. The Lennard-Jones interactions were cut off at 1.2 nm, and the forces were smoothly switched to zero between 1.0 nm and 1.2 nm. The smooth particle mesh Ewald (PME) method [34] with a cut-off of 1.2 nm was employed for electrostatic interactions. The Nosé–Hoover thermostat [35, 36] with a time constant of 1 ps was used

to keep the temperatures of the protein, the membrane, and the solvent separately at 310 K (Set 2) or 343 K (Set 3). All membranes were fluid at the simulated temperatures. The semi-isotropic Parrinello–Rahman barostat [25] with a time constant of 5 ps was employed to keep the pressure at 1 bar. The bonds involving hydrogens were constrained using LINCS [37], while SETTLE [38] was employed to constrain the geometry of water molecules.

Simulations were run using GROMACS v.5.0.x [26] using a time step of 2 fs.

A.4 All-Atom Simulations of the Solvation of Proteins by DHA

We also employed all-atom simulations to study whether certain protein types are more prone to be solvated by DHA. To this end, we simulated four structurally different transmembrane proteins: 1) the transmembrane domain of the human receptor tyrosine kinase (ErbB1, PDB id: 2M0B), a single helix; 2) a dimer formed by two Glycophorin A peptides [39] (GpA dimer, PDB id: 1AFO); 3) $A_{2A}R$ (PDB id: 3EML) [2], a heptahelical bundle employed in the CG free energy calculation; and 4) the voltage-dependent anion channel (VDAC, PDB id: 3EMN) [40], a β-barrel. Notably, this selection of proteins provided an increase in transmembrane helix count (1, 2, and 7), and included one protein formed by β sheets. All of these proteins were embedded in a lipid bilayer consisting of a total of 250 lipids with equimolar concentrations of CHOL, dipalmitoyl-phosphatidylcholine (DPPC, two saturated chains; di-16:0), DOPC (two monounsaturated chains; di-18:1), dilinoleoyl-phosphatidylcholine (DLiPC, two diunsaturated chains; di-18:2), and stearoyl-docosahexaenoyl-phosphatidylcholine (SDPC, one saturated 18:0 chain and one polyunsaturated 22:6 chain (DHA)). Here, SDPC was used instead of SDPE to exclude a potential effect of different lipid head groups. The lipid composition contains equal amounts of saturated palmitic acid, monounsaturated oleic acid and diunsaturated linoleic acid. Additionally, the polyunsaturated docosahexaenoic acid and saturated stearic acid are provided by the two chain types of SDPC. This approach of using only lipids that had the same head group allowed us to explore the influence of the saturation level (with 0, 1, 2, or 6 double bonds) in a controlled manner. The bilayers were solvated by 45 waters per lipid, and $\sim$150 mM of NaCl was included together with additional counter ions to neutralize protein charge. The proteins are depicted in the middle column and the systems in the rightmost column of Fig. 5 in the main text.

For the the GPCR $A_{2A}R$ helix 8, not part of the transmembrane helix bundle, was ommitted from the COM definition in the RDF plots shown in the leftmost column of Fig. 5 in the main paper. For the GpA dimer, due to its shape, the RDFs were calculated separately for the monomers and the average value is reported.

The input structures for GROMACS were generated [33] using the Membrane Builder [29] of the CHARMM-GUI website [32], and the systems were simulated for 4 µs using the input parameters provided by CHARMM-GUI [33] (see previous Subsection for details).

B Supplementary Results

B.1 The L_o–L_d Transition is Smooth

Using the protein-free systems, we verified that the performed alchemical transformation DSPC→DOPC indeed results in a change of membrane phase from L_o to L_d when the coarse-grained Martini force field is used. This change is evident in final structures of the systems corresponding to DSPC/cholesterol (L_o) and DOPC/cholesterol (L_d) systems, shown on the left and right in Fig. A. The transformation at fixed temperature is possible due to the limited representation of phase transitions in the Martini model. Namely, DSPC begins its transition to the gel phase at 294 K [8], while the experimental main transition temperature is 328 K. This allows us to run both DOPC/cholesterol and DSPC/cholesterol at a reasonable temperature of 315 K and still produce the two distinct liquid phases at the endpoints of the alchemical transformation path.

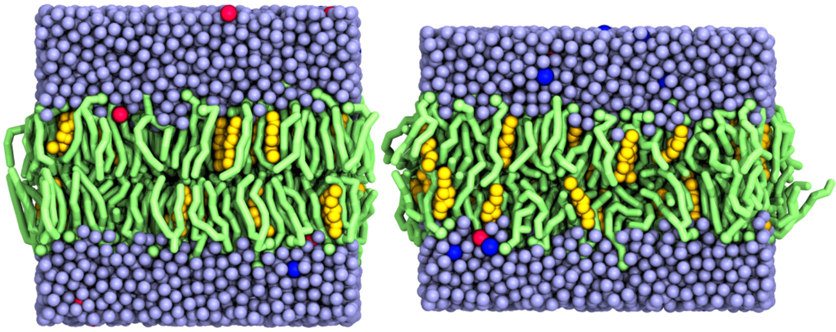

Fig A. Snapshots of the protein- and SDPE-free DSPC/cholesterol (L_o, left) and DOPC/cholesterol (L_d, right) systems, *i.e.* the endpoints ($\lambda = 0/1$) of the alchemical transformation. DSPC/DOPC is shown in green, cholesterol in yellow, water beads in light blue, and ions in red and blue.

Quantitatively, the decrease in average order parameter (see bottom panel of Fig. 2A in the main text) and increase in area per lipid (see bottom panel of Fig. B) as well as the increase in diffusion coefficient (see Fig. C) confirm that the change of phase takes place as expected. These data are extracted from the last 450 ns of the 500 ns protein-free simulations. Importantly, the area per lipid and order parameter profiles are continuous indicating adequate sampling of intermediate λ states, and hence likely convergence in the terms in Eq (4).

Bottom panel of Fig. 2A in the main text shows that the alchemical transformation takes the membrane smoothly from the L_o to the L_d phase. The 2nd rank acyl chain order parameter (S_n), averaged over all lipid chains and over simulation time, shows a continuous change from L_o-like behavior to L_d-like acyl chain order. A continuous change can also be observed for the area per lipid and the lateral diffusion coefficients of lipids, shown in Figs. B and C, respectively. The DSPC/CHOL system ($\lambda = 0$) is ordered but in the fluid phase; there are no sudden changes related to phase transitions in any of the studied quantities. Moreover, a gel phase system (pure DSPC at 285 K [8]) has much higher order (0.81), lower area per lipid (48 Å^2), and smaller diffusion coefficient (0.1×10^{-7} cm^2/s) than the DSPC/CHOL system, indicating that the DSPC/CHOL mixture is in the L_o phase. The presence of CHOL in the DOPC/CHOL ($\lambda = 1$) system, on the other hand, does not induce formation of the L_o phase. Instead, the behavior of the DOPC/CHOL system is essentially similar to a CHOL-free L_d phase (pure DOPC at 315 K), which provides an average order parameter of 0.31, an area per lipid of 69 Å^2, and a diffusion coefficient of 5×10^{-7} cm^2/s. The presence of DHA has a

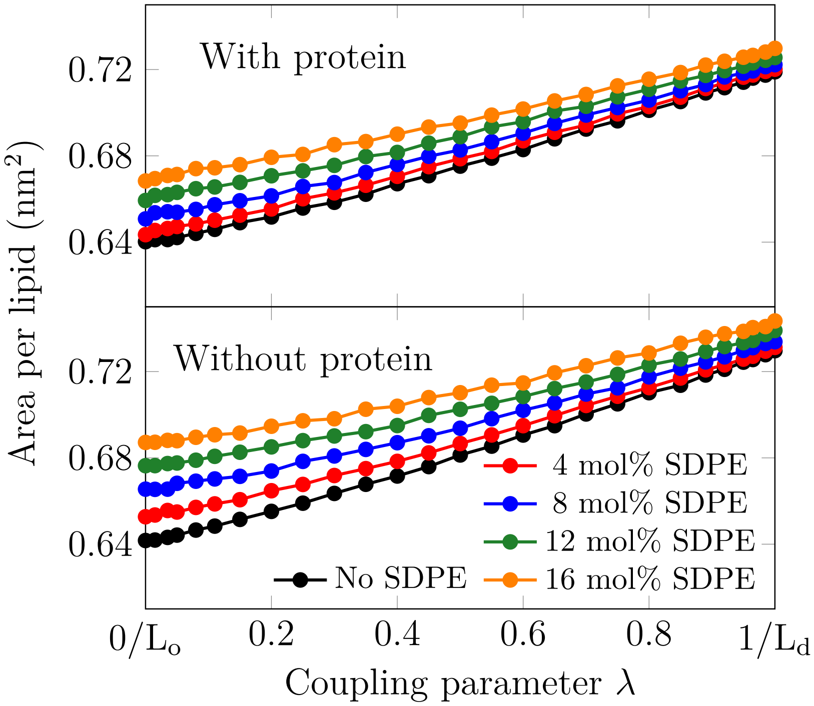

Fig B. Area per phospholipid (including SDPE) as a function of the coupling parameter λ in the protein-containing and the protein-free systems. The protein area, estimated as a difference of the total areas of the protein-containing and protein-free L_o system simulation, is subtracted from the curves of the protein-containing system. Error bars showing standard error are indistinguishable from the markers.

small effect on the properties of the L_o phase, slightly increasing its area per lipid (see Fig. B) and reducing chain order (see Fig. 2A in the main text).

Additionally, we also considered partitioning between membranes whose compositions resembled those observed in experiments on model membranes (see systems marked with * in Table A). The order parameters as a function of coupling parameter λ are shown for the protein-free membranes in Fig. D. It is clear that while there is a decline in membrane order upon the change of part of the DSPC lipids into DOPC ones, the two phases are structurally less different than the ones containing only DSPC or DOPC at a time.

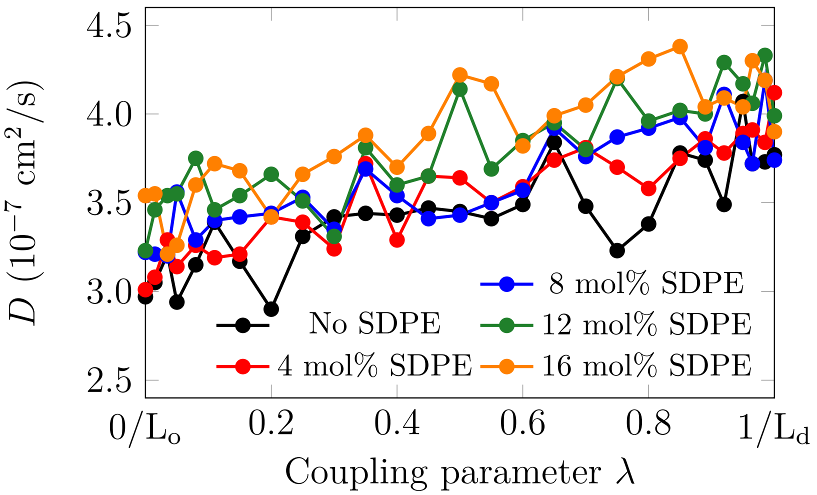

Fig C. Lateral diffusion coefficients of the mutated lipid (DSPC→DOPC) as a function of the coupling parameter λ in the protein-free system. The motion of lipids is analyzed with respect to the center of mass of the whole bilayer. The diffusion coefficient is extracted as a linear fit to the calculated mean squared displacement data in the lag time interval from 10 ns to 50 ns. Lipid mobility seems to increase by ∼30% upon phase transition. SDPE does not seem to have a strong effect on lipid mobility. Standard error is not shown since reliable estimates for the diffusion coefficient cannot be extracted from blocks that are much shorter than the entire simulation.

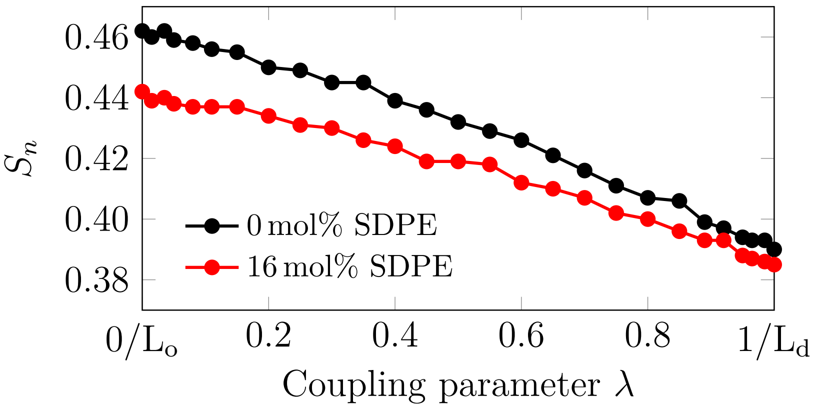

Fig D. Average 2nd rank acyl chain order parameter (S_n) of phosphatidylcholines (DSPC, DOPC, and the mutated intermediate) in the systems with a realistic composition in the L_d and L_o phases and in the absence of any proteins.

B.2 Saturation of the $A_{2A}R$ Surface by DHA

The RDFs of different lipid chains in the coarse-grained simulations, as shown in Fig. 4A in the main paper for the system with 4% SDPE, are shown in Fig. E for all simulated SDPE concentrations.

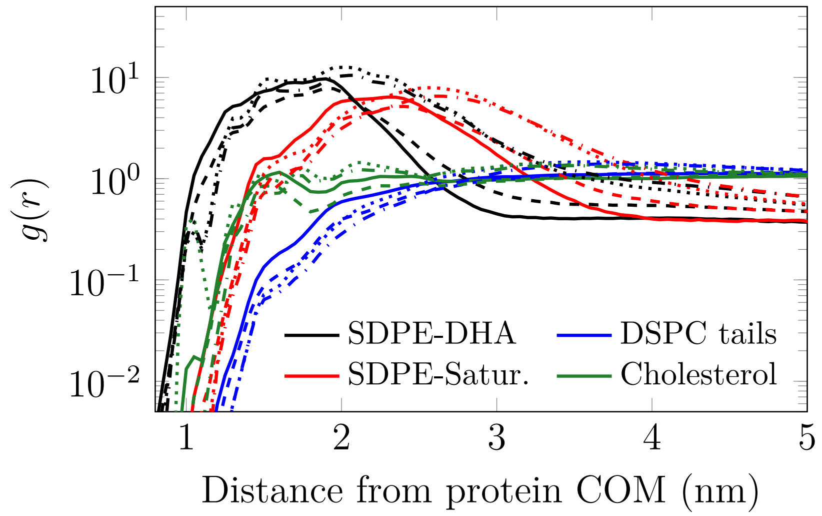

Fig E. Radial distribution functions of different lipid chains around the $A_{2A}R$ center of mass systems with 4 mol% (solid lines), 8 mol% (dashed lines), 12 mol% (dotted lines), and 16 mol% (dashdotted lines) of SDPE. Data are extracted from the last 5 µs of the 10 µs simulations. Error bars are small (see data for the system with 4% SDPE in Fig. 4A with the main paper), and are hence not shown for clarity.

Additionally, we also evaluated the RDFs of different lipid chains around the D_2R and GLUT1 proteins in the coarse-grained scheme, shown in Fig. F. The tendency of DHA to solvate these multi-pass helical bundles is evident, in line with the findings from all-atom simulations described in the main paper.

We evaluated the stability of the solvation shell in the coarse-grained simulations with the exchange rates of SDPE and cholesterol at the $A_{2A}R$ surface as follows: First, we checked for each molecule of the chosen type whether they were in contact with the receptor. A contact was registered if the chosen bead (hydroxyl "ROH" for cholesterol and phosphate "PO4" for SDPE) was within 0.8 nm of any protein bead. Next, for each molecule the time evolution of the contacts (with values 1 or 0) was autocorrelated and averaged over all molecules of the chosen type. Finally, the obtained autocorrelation curves were fitted with a double exponential function with two characteristic time constants τ_1 and τ_2. These values are given in Table B. Notably, the prefactor of the exponential term with the larger time constant τ_2 was found to dominate the fit.

The number of contacts between DHA chains (normalized by the number of beads in the DHA chains) and $A_{2A}R$ observed in the coarse-grained simulations is shown in Fig. G for different SDPE concentrations and for the different windows along the alchemical transformation. Here, a contact is defined by a cut off of 0.6 nm, and the last 500 ns of the free energy windows is included in the analysis.

These curves demonstrate two interesting effects. First, the number of DHA–$A_{2A}R$ contacts decrease towards the L_d phase indicating a fair competition between the polyunsaturated and the monounsaturated chains toward the protein surface. This suggests that the SDPE corona dissolves in the L_d phase. Second, the number of contacts decreases upon increasing SDPE concentration. This indicates that the surface

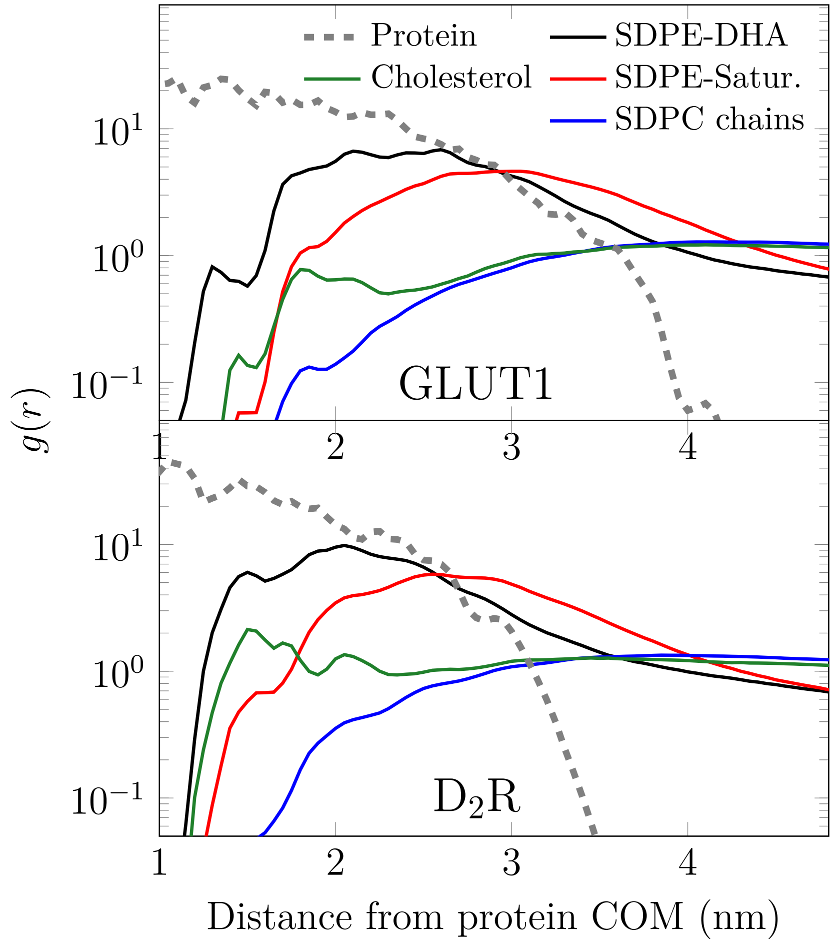

Fig F. Radial distribution functions of different lipid chains around the D_2R and GLUT1 centers of mass in systems with 16 mol% SDPE. Data are extracted from the last 5 µs of the 10 µs simulations.

of $A_{2A}R$ saturates at a fairly low SDPE concentration. Due to this saturation, an increasing amount of DHA is left in the bulk membrane, where it perturbs the membrane properties (see Figs. B, C, D, and 2A in the main text). However, the amount of free SDPE is small enough so that it does not phase separate from the ordered phase in the studied systems (data not shown).

The saturation of the $A_{2A}R$ surface in the coarse-grained simulations is further evidenced by Fig. H. The black markers show the number of SDPE molecules whose phosphate bead is within 0.6 nm from the $A_{2A}R$ surface. This value is fitted well with an exponential function that saturates towards a value of 25.4 indicating that on average ∼13 SDPE molecules can fit to the protein surface in both membrane leaflets.

The blue bars in Fig. H show the SDPE concentration in the bulk L_o membrane as a function of the overall concentration of SDPE. This value is calculated as the ratio of the free SDPE (total number of SDPE − the number of protein-bound SDPE) and the total number of free lipids (400 − the number of protein-bound SDPE).

As expected, the bulk concentration of SDPE does not grow linearly with increasing total SDPE concentration. The data at high (12–16 mol%) SDPE concentration shows a slope of ∼1 indicating that all added SDPE goes to the bulk membrane. A similar fit to

Table B. Exchange time constants of cholesterol and SDPE at the protein surface. For systems marked with *, the data were extracted from the last 5 μs of the 10 μs equilibration simulations during which the DHA shell formed. For other systems, the data were extracted from the free energy windows that were 1 μs long.

System	Cholesterol		SDPE	
	τ_1 (ns)	τ_2 (ns)	τ_1 (ns)	τ_2 (ns)
No SDPE (L_o)	2	61	–	–
4 mol% SDPE (L_o)*	31	323	2	209
8 mol% SDPE (L_o)*	11	138	9	203
12 mol% SDPE (L_o)*	7	98	6	152
16 mol% SDPE (L_o)*	10	103	8	129
0 mol% SDPE (L_d)	1	49	–	–
4 mol% SDPE (L_d)	3	46	2	75
8 mol% SDPE (L_d)	2	46	1	55
12 mol% SDPE (L_d)	2	43	1	53
16 mol% SDPE (L_d)	2	33	2	57

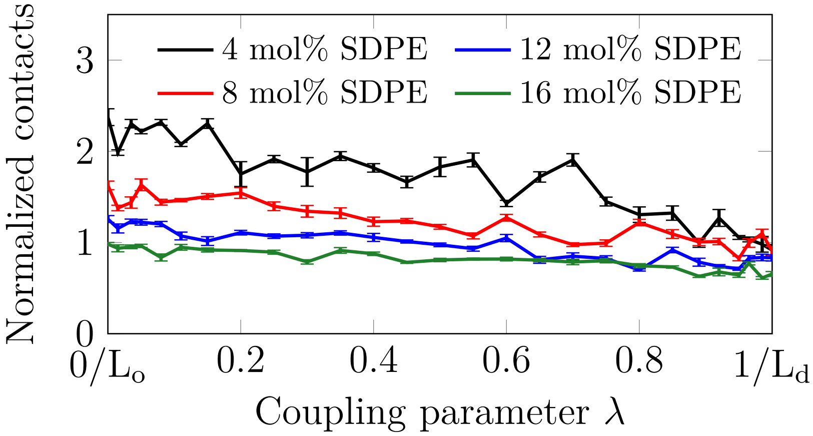

Fig G. Number of contacts between the DHA and the protein upon the alchemical transformation DSPC→DOPC. The numbers are normalized by the number of DHA beads present. Error bars show standard error.

the data at low (0–4 mol%) SDPE concentration provides a much smaller slope of ∼0.6 suggesting that half of the added SDPE participates in the formation of the SDPE corona around $A_{2A}R$. These two slopes cross at an SDPE concentration of ∼6%. Hence, at around this concentration, corresponding to 12 SDPE molecules per leaflet in our simulations, the saturation is complete. This value agrees well with the estimate of surface saturation (13) given above.

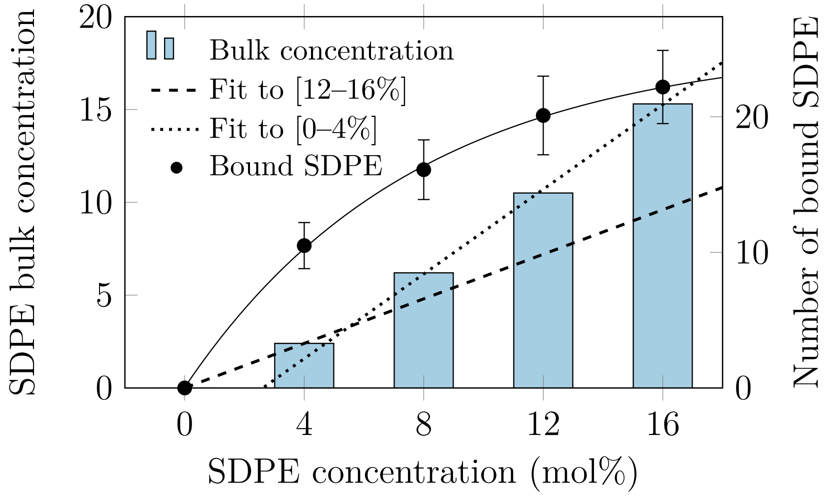

Fig H. The saturation of the $A_{2A}R$ surface by SDPE in the L_o phase membrane (DSPC/CHOL). The figure shows both the concentration of free SDPE in the bulk membrane as a function of total SDPE concentration (with linear fits) as well as the number of protein-bound SDPE as a function of SDPE concentration.

B.3 Spatially Resolved Effects of SDPE and $A_{2A}R$ on Membrane Order

As shown in Fig. 2 in the main text as well as in Fig. I, both $A_{2A}R$ and SDPE reduce the order of the membrane; $A_{2A}R$ in its vicinity and SDPE in the entire membrane. However, in the immediate vicinity of the receptor, the order parameters are similar in the absence and presence of SDPE. This is also demonstrated by Fig. J, which shows the average order parameter values as a function of distance from the receptor center of mass. These results further demonstrate the finding based on Fig. 2B in the main text that the presence of $A_{2A}R$ is able to compensate for the disordering effect of SDPE and *vice versa* through their mutual association. This limits the perturbation caused by the receptor on the L_o phase and hence renders $A_{2A}R$ more compatible with the L_o phase.

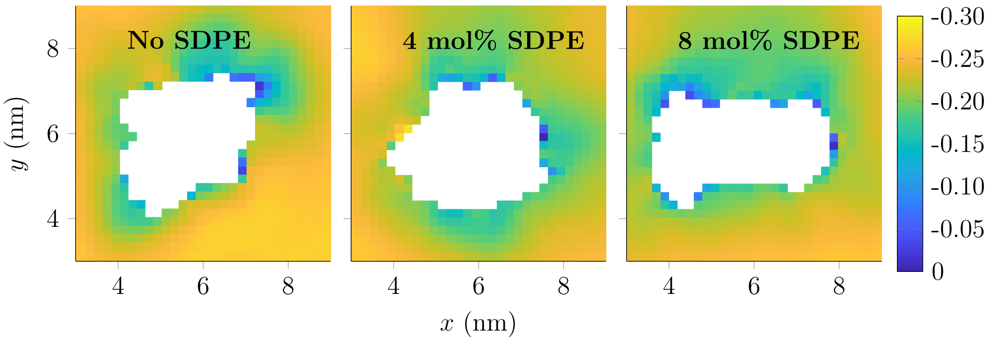

Fig I. Spatial variation of the average deuterium order parameter. The average deuterium order parameter (S_{CD}) is extracted from all-atom simulations with 0 (left), 4 (middle), and 8 mol% (right) SDPE. Only data near the protein are shown. Averaging of the data was performed over both *sn*-1 and *sn*-2 chains, over both membrane leaflets, and over time. The data shown here were binned on a grid (with a bin width of 0.2 nm), using the `g_lomepro` tool [41]. Due to the limitations of this tool, protein is centered prior to the analysis, but no rotational fit is performed. Therefore, the protein shape appears to be different between the plots. For data averaged and projected to one dimension, see Fig. J.

The average deuterium order parameter of stearic acid chains (two in DSPC, one in SDPE) in the fine-grained all-atom simulations is shown as a function of distance from $A_{2A}R$ center of mass (COM) in Fig. J. This figure shows how the order parameter grows faster as a function of distance from $A_{2A}R$ COM when the membrane contains no SDPE. Importantly, the result agrees well with the order parameter values in Fig. 2 in the main paper as well as in Fig. I, which showed average values of approximately $-S_{CD} = -0.24$.

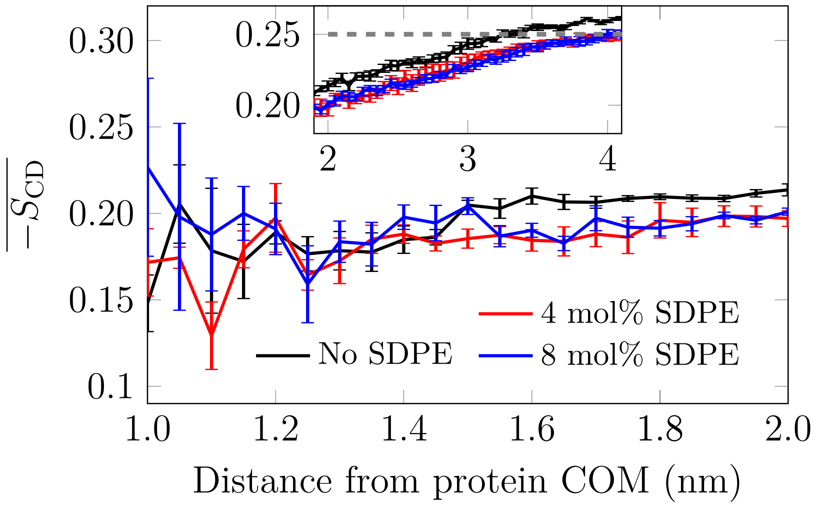

Fig J. Average deuterium order parameter of stearic acid chains as a function of distance from $A_{2A}R$ COM extracted from the fine-grained (all-atom) simulations. Inset shows data further away from the receptor. Error bars show standard error calculated by splitting the trajectory into 5 pieces.

B.4 The Presence of SDPE does not Affect Hydrophobic Mismatch

The data for membrane thickness as a function of distance from the protein surface are shown in Fig. K. These data are obtained from the coarse-grained systems with SDPE concentrations of 0 mol%, 4 mol%, and 8 mol% in both L_o and L_d phases. Here thickness is defined as the local inter-leaflet phosphate bead distance. The maps are calculated in a grid of 61×61 points using the `g_lomepro` tool [41]. The position of a protein was defined by a 2D density map. Then, for each grid point the shortest distance to the protein was calculated, and these distances were binned at a ∼0.1 nm spacing. For each bin, the mean thickness was calculated. The trajectory was analyzed in 5 parts and the mean and standard error of the calculated profiles are shown.

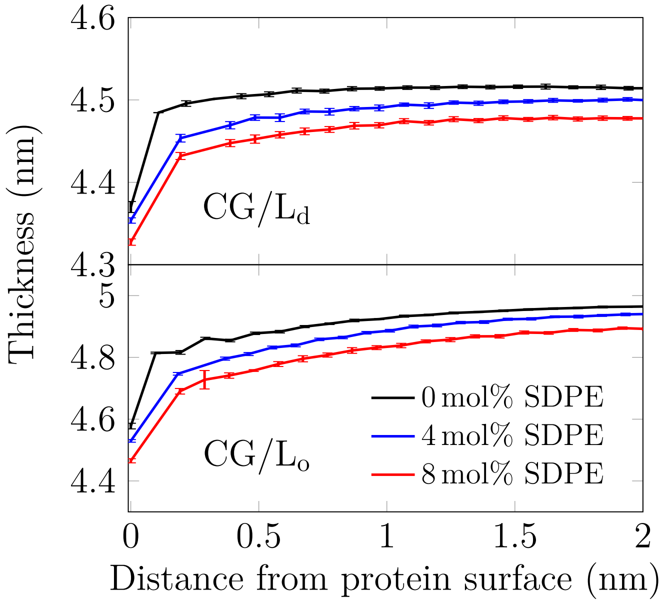

Fig K. Membrane thickness as a function of distance from the protein surface for all-atom systems containing 0 mol% (A), 4 mol% (B), and 8 mol% (C) of SDPE in the coarse-grained simulations. Error bars show standard error calculated by splitting the trajectory into 5 pieces.

The data in Fig. K indicate that the hydrophobic mismatch of $A_{2A}R$ in the L_o phase is essentially unaffected by SDPE. Hence, negation of hydrophobic mismatch is not the mechanism by which SDPE renders $A_{2A}R$ more compatible with the L_o phase.

B.5 SDPE Shell Does Not Increase the Conformation Entropy of the Receptor

The root mean squared fluctuations (RMSF) of each $A_{2A}R$ residue in the CG simulations are shown in Fig. L. These data, calculated using the `gmx rmsf` tool of GROMACS, are shown for 0, 4, and 8 mol% of SDPE in both L_o and L_d phases.

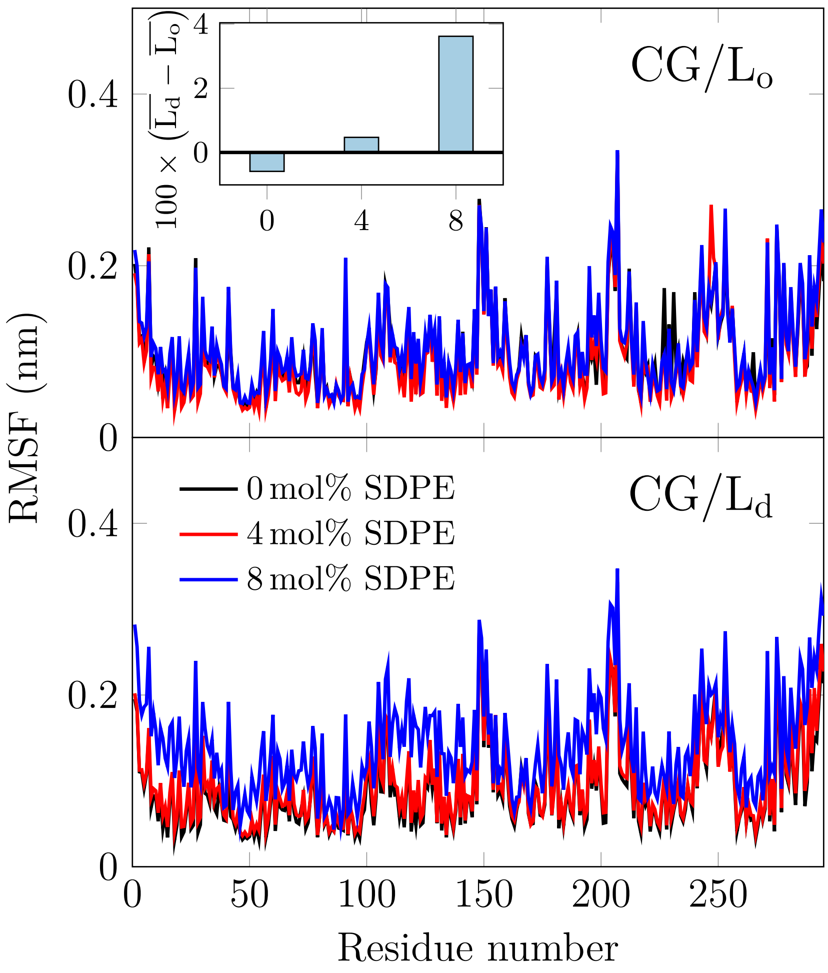

Fig L. RMSF of each residue in the CG simulations with 0, 4, or 8 mol% of SDPE in both L_o and L_d phases. The inset shows the difference in mean RMSF values between L_o and L_d phases as a function of SDPE concentration.

The curves in Fig. L demonstrate that the protein has more conformational freedom in membranes with SDPE. However, upon the addition of SDPE, the values in the L_d phase grow more, suggesting that conformational entropy of $A_{2A}R$ drives it to the L_d phase as SDPE concentration is increased. As our free energy calculations show that the reverse is true, increase in conformational entropy due to the SDPE shell is not the factor behind increased partitioning of $A_{2A}R$ to the L_o phase upon an increase in SDPE concentration.

B.6 Convergence of the All-Atom Solvation Simulations

The time evolution of contacts between the proteins and the different lipid chains in the all-atom solvation simulations is shown in Fig. M. The data are normalized by the number of similar chains (one for cholesterol). While other proteins show no clear preference for any chain type, the preference of $A_{2A}R$ for DHA is apparent.

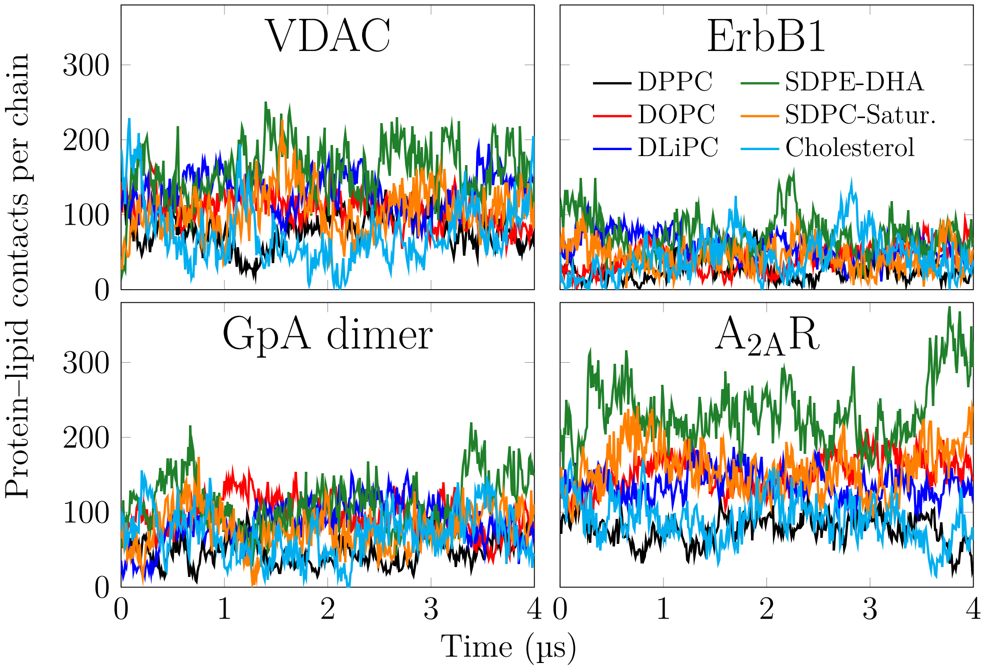

Fig M. Time evolution of protein–lipid chain contacts in all-atom solvation simulations.

B.7 Methodological Limitations

Extracting free energies of transfer from MD simulations is a difficult task. Currently, all-atom models cannot provide enough sampling, so coarse-grained models are often preferred in such studies [1, 42–45].

One option is to simulate a membrane with coexisting phases and use umbrella sampling to extract the free energy of transfer between the domains. This approach has also been recently employed [44]. However, there are some issues with this approach: The reaction coordinate is often poorly determined as the domain boundaries are not fixed, and strong hysteresis effects are possible. Moreover, the compositions of the coexisting phases in the Martini model do not reflect reality as the domain boundaries are too sharp and the compositions of the phases are too exclusive: The L_o phase contains almost solely the lipids with saturated chains and cholesterol, while the L_d phase consists of the lipid with unsaturated chains only.

Another option is to simulate such coexisting phases and study the spontaneous protein partitioning therein, as recently reported in Ref. 42. Moreover, the free energy of transfer can also be estimated from the densities of the protein in the two phases. However, in such simulations the sampling of the L_o phase is often poor. This results at least partly from the fact that at the simulation temperatures commonly used to study such phase coexistence (∼295 K), the L_o phase is almost solid in the coarse-grained Martini model.

For small molecules — such as lipids or single-pass peptides partitioning between different membrane domains or phases — the free energy of transfer can be calculated by making the lipid or peptide disappear from these separate domains in two sets of simulations. The difference between these values provides the free energy of transfer. This approach has recently been used in, *e.g.*, Ref. [1]. Unfortunately, this approach is not applicable to larger objects such as multi-pass proteins since making a large object disappear within the membrane would lead to poor convergence.

The approach introduced in this study for extracting free energies of transfer through free energy perturbation overcomes many issues of the previous approaches. The protein samples both phases carefully and there is no hysteresis. Moreover, the reaction coordinate is well-defined, and at the used temperature of 315 K, both phases are fluid. Our method also allows us to study partitioning between phases enriched in lipids with saturated chains (such as DSPC) and those enriched in lipids with two mono-unsaturated chains (such as DOPC). This DSPC/DOPC/CHOL mixture undergoes phase separation in experiments [46]. However, it is not possible to use this in the coexistence approach, since at the used size scales such compositions do not undergo phase separation — likely due to the large line tension between the respective phases. Moreover, we can fine-tune the compositions of the more ordered and less ordered phases based on the tie lines measured experimentally. This is what we have done when we used the "realistic" membrane compositions in this work.

One possible issue with our approach is that cholesterol is present in both phases at equal amounts. This is likely somewhat off of reality, yet in ternary mixtures of lipids with monounsaturated and saturated chains with cholesterol, the tie lines indicate that the concentrations of cholesterol in the coexisting phases are not very different [47, 48].

Another issue is the solubility of PUFA-chained hybrid lipids in coexisting ordered and disordered membrane regions. For mixtures containing DHA-chained hybrid lipids, no tie lines have unfortunately been measured, yet the solubility of SDPC to the ordered phase has been estimated to be similar to that of DOPC [49]. Therefore, the ratio of PUFA-chained lipids in the disordered and ordered phases is likely 2-to-1, or even less. Moreover, in the brain cortex tissue, the amounts of DHA in raft and non-raft regions are not very different [14].

Finally, we also note that the values calculated using the coarse-grained scheme are

large, yet this is a general trend in such studies (see *e.g.* Refs. 1, 44, and 43). Finally, the calculation of the values obtained for single-pass WALP peptides here (see Section S1.1) and in Ref. [1] highlight that our method does not overestimate the free energies of transfer.

B.8 Effect of DHA-containing Lipid Head Group on Partitioning

We repeated the free energy of transfer calculation in the coarse-grained scheme for $A_{2A}R$ in the realistic membrane setting using a mixture in which SDPE was replaced by SDPC. At 16 mol% SDPE, the L_o phase was favored by 4.4 kJ/mol (see Fig. 3B in the main paper), whereas at the similar concentration of SDPC, the free energy of transfer was −0.2 kJ/mol, indicating no specific tendency towards either phase. Hence, SDPE seems to be slightly better in promoting partitioning into the L_o phase, yet such small differences are likely within the errors due to the limited descriptions of lipid chemistry in the coarse-grained scheme.

References

1. Schäfer LV, de Jong DH, Holt A, Rzepiela AJ, de Vries AH, Poolman B, et al. Lipid Packing Drives the Segregation of Transmembrane Helices into Disordered Lipid Domains in Model Membranes. Proc Natl Acad Sci USA. 2011;108(4):1343–1348.

2. Jaakola VP, Griffith MT, Hanson MA, Cherezov V, Chien EY, Lane JR, et al. The 2.6 Angstrom Crystal Structure of a Human A_{2A} Adenosine Receptor Bound to an Antagonist. Science. 2008;322(5905):1211–1217.

3. Chen CT, Liu Z, Ouellet M, Calon F, Bazinet RP. Rapid β-Oxidation of Eicosapentaenoic Acid in Mouse Brain: An in Situ Study. Prostaglandins, Leukot Essent Fatty Acids. 2009;80(2):157–163.

4. Marrink SJ, Risselada HJ, Yefimov S, Tieleman DP, De Vries AH. The MARTINI Force Field: Coarse Grained Model for Biomolecular Simulations. J Phys Chem B. 2007;111(27):7812–7824.

5. Monticelli L, Kandasamy SK, Periole X, Larson RG, Tieleman DP, Marrink SJ. The MARTINI Coarse-grained Force Field: Extension to Proteins. J Chem Theory Comput. 2008;4(5):819–834.

6. de Jong DH, Singh G, Bennett WD, Arnarez C, Wassenaar TA, Schäfer LV, et al. Improved Parameters for the Martini Coarse-Grained Protein Force Field. J Chem Theory Comput. 2012;9(1):687–697.

7. Periole X, Cavalli M, Marrink SJ, Ceruso MA. Combining an Elastic Network with a Coarse-Grained Molecular Force Field: Structure, Dynamics, and Intermolecular Recognition. J Chem Theory Comput. 2009;5(9):2531–2543.

8. Rodgers JM, Sørensen J, de Meyer FJM, Schiøtt B, Smit B. Understanding the Phase Behavior of Coarse-Grained Model Lipid Bilayers through Computational Calorimetry. J Phys Chem B. 2012;116(5):1551–1569.

9. Huang T, Lee C, Das Gupta S, Blume A, Griffin R. A Carbon-13 and Deuterium Nuclear Magnetic Resonance Study of Phosphatidylcholine/Cholesterol Interactions: Characterization of Liquid-Gel Phases. Biochemistry. 1993;32(48):13277–13287.

10. Bennett CH. Efficient Estimation of Free Energy Differences from Monte Carlo Data. J Comput Phys. 1976;22(2):245–268.

11. Beaulieu JM, Gainetdinov RR. The Physiology, Signaling, and Pharmacology of Dopamine Receptors. Pharmacol Rev. 2011;63(1):182–217.

12. Müller CP, Reichel M, Mühle C, Rhein C, Gulbins E, Kornhuber J. Brain Membrane Lipids in Major Depression and Anxiety Disorders. BBA-Mol Cell Biol L. 2015;1851(8):1052–1065.

13. Martín V, Fabelo N, Santpere G, Puig B, Marín R, Ferrer I, et al. Lipid Alterations in Lipid Rafts from Alzheimer's Disease Human Brain Cortex. J Alzheimers Dis. 2010;19(2):489–502.

14. Fabelo N, Martín V, Santpere G, Marín R, Torrent L, Ferrer I, et al. Severe Alterations in Lipid Composition of Frontal Cortex Lipid Rafts from Parkinson's Disease and Incidental Parkinson's Disease. Mol Med. 2011;17(9):1107–1118.

15. du Bois TM, Deng C, Huang XF. Membrane Phospholipid Composition, Alterations in Neurotransmitter Systems and Schizophrenia. Prog Neuropsychopharmacol Biol Psychiatry. 2005;29(6):878–888.

16. Guixà-González R, Javanainen M, Gómez-Soler M, Cordobilla B, Domingo JC, Sanz F, et al. Membrane Omega-3 Fatty Acids Modulate the Oligomerisation Kinetics of Adenosine A_{2A} and Dopamine $_2$ Receptors. Sci Rep. 2016;6:19839.

17. Deng D, Xu C, Sun P, Wu J, Yan C, Hu M, et al. Crystal Structure of the Human Glucose Transporter GLUT1. Nature. 2014;510(7503):121–125.

18. Ximenes da Silva A, Lavialle F, Gendrot G, Guesnet P, Alessandri JM, Lavialle M. Glucose Transport and Utilization Are Altered in the Brain of Rats Deficient in N-3 Polyunsaturated Fatty Acids. J Neurochem. 2002;81(6):1328–1337.

19. Pifferi F, Roux F, Langelier B, Alessandri JM, Vancassel S, Jouin M, et al. (N-3) Polyunsaturated Fatty Acid Deficiency Reduces the Expression of Both Isoforms of the Brain Glucose Transporter GLUT1 in Rats. J Nutr. 2005;135(9):2241–2246.

20. Pifferi F, Jouin M, Alessandri J, Haedke U, Roux F, Perriere N, et al. N-3 Fatty Acids Modulate Brain Glucose Transport in Endothelial Cells of the Blood–Brain Barrier. Prostaglandins Leukot Essent Fatty Acids. 2007;77(5-6):279–286.

21. Uppamoochikkal P, Tristram-Nagle S, Nagle JF. Orientation of Tie-Lines in the Phase Diagram of Dopc/Dppc/Cholesterol Model Biomembranes. Langmuir. 2010;26(22):17363–17368.

22. de Jong DH, Baoukina S, Ingólfsson HI, Marrink SJ. Martini Straight: Boosting Performance Using a Shorter Cutoff and GPUs. Comput Phys Comm. 2016;199:1–7.

23. Páll S, Hess B. A Flexible Algorithm for Calculating Pair Interactions on SIMD Architectures. Comput Phys Commun. 2013;184(12):2641–2650.

24. Bussi G, Donadio D, Parrinello M. Canonical Sampling through Velocity Rescaling. J Chem Phys. 2007;126(1):014101.

25. Parrinello M, Rahman A. Polymorphic Transitions in Single Crystals: A New Molecular Dynamics Method. J Appl Phys. 1981;52(12):7182–7190.

26. Abraham MJ, Murtola T, Schulz R, Páll S, Smith JC, Hess B, et al. GROMACS: High Performance Molecular Simulations through Multi-Level Parallelism from Laptops to Supercomputers. SoftwareX. 2015;1:19–25.

27. Wassenaar TA, Pluhackova K, Böckmann RA, Marrink SJ, Tieleman DP. Going Backward: A Flexible Geometric Approach to Reverse Transformation from Coarse Grained to Atomistic Models. J Chem Theory Comput. 2014;10(2):676–690.

28. Mabrey S, Sturtevant JM. Investigation of Phase Transitions of Lipids and Lipid Mixtures by Sensitivity Differential Scanning Calorimetr. Proc Natl Acad Sci USA. 1976;73(11):3862–3866.

29. Wu EL, Cheng X, Jo S, Rui H, Song KC, Dávila-Contreras EM, et al. CHARMM-GUI Membrane Builder toward Realistic Biological Membrane Simulations. J Comput Chem. 2014;35(27):1997–2004.

30. Klauda JB, Venable RM, Freites JA, O'Connor JW, Tobias DJ, Mondragon-Ramirez C, et al. Update of the CHARMM All-Atom Additive Force Field for Lipids: Validation on Six Lipid Types. J Phys Chem B. 2010;114(23):7830–7843.

31. Best RB, Zhu X, Shim J, Lopes PE, Mittal J, Feig M, et al. Optimization of the Additive CHARMM All-Atom Protein Force Field Targeting Improved Sampling of the Backbone ϕ, ψ and Side-Chain $\chi1$ and $\chi2$ Dihedral Angles. J Chem Theory Comput. 2012;8(9):3257–3273.

32. Jo S, Kim T, Iyer VG, Im W. CHARMM-GUI: A Web-Based Graphical User Interface for CHARMM. J Comput Chem. 2008;29(11):1859–1865.

33. Lee J, Cheng X, Swails JM, Yeom MS, Eastman PK, Lemkul JA, et al. CHARMM-GUI Input Generator for NAMD, GROMACS, AMBER, OpenMM, and CHARMM/OpenMM Simulations Using the CHARMM36 Additive Force Field. J Chem Theory Comput. 2015;12(1):405–413.

34. Essmann U, Perera L, Berkowitz ML, Darden T, Lee H, Pedersen LG. A Smooth Particle Mesh Ewald Method. J Chem Phys. 1995;103(19):8577–8593.

35. Nosé S. A Unified Formulation of the Constant Temperature Molecular Dynamics Methods. J Chem Phys. 1984;81(1):511–519.

36. Hoover WG. Canonical Dynamics: Equilibrium Phase-Space Distributions. Phys Rev A. 1985;31(3):1695–1697.

37. Hess B, Bekker H, Berendsen HJ, Fraaije JG. LINCS: A Linear Constraint Solver for Molecular Simulations. J Comput Chem. 1997;18(12):1463–1472.

38. Miyamoto S, Kollman PA. Settle: An Analytical Version of the SHAKE and RATTLE Algorithm for Rigid Water Models. J Comput Chem. 1992;13(8):952–962.

39. MacKenzie KR, Prestegard JH, Engelman DM. A Transmembrane Helix Dimer: Structure and Implications. Science. 1997;276(5309):131–133.

40. Ujwal R, Cascio D, Colletier JP, Faham S, Zhang J, Toro L, et al. The Crystal Structure of Mouse VDAC1 at 2.3 Å Resolution Reveals Mechanistic Insights into Metabolite Gating. Proc Natl Acad Sci USA. 2008;105(46):17742–17747.

41. Gapsys V, de Groot BL, Briones R. Computational Analysis of Local Membrane Properties. J Comput Aid Mol Des. 2013;27(10):845–858.

42. de Jong DH, Lopez CA, Marrink SJ. Molecular View on Protein Sorting into Liquid-Ordered Membrane Domains Mediated by Gangliosides and Lipid Anchors. Farad Discuss. 2013;161:347–363.

43. Lin X, Gorfe AA, Levental I. Protein Partitioning into Ordered Membrane Domains: Insights from Simulations. Biophys J. 2018;114(8):1936–1944.

44. Lorent JH, Diaz-Rohrer B, Lin X, Spring K, Gorfe AA, Levental KR, et al. Structural Determinants and Functional Consequences of Protein Affinity for Membrane Rafts. Nat Commun. 2017;8(1):1219.

45. Ackerman DG, Feigenson GW. Effects of Transmembrane α-Helix Length and Concentration on Phase Behavior in Four-Component Lipid Cixtures: a Molecular Dynamics Study. J Phys Chem B. 2016;120(17):4064–4077.

46. Zhao J, Wu J, Heberle FA, Mills TT, Klawitter P, Huang G, et al. Phase Studies of Model Biomembranes: Complex Behavior of DSPC/DOPC/cholesterol. BBA-Biomembranes. 2007;1768(11):2764–2776.

47. Veatch S, Polozov I, Gawrisch K, Keller S. Liquid Domains in Vesicles Investigated by NMR and Fluorescence Microscopy. Biophys J. 2004;86(5):2910–2922.

48. Veatch SL, Soubias O, Keller SL, Gawrisch K. Critical Fluctuations in Domain-Forming Lipid Mixtures. Proc Natl Acad Sci USA. 2007;104(45):17650–17655.

49. Konyakhina TM, Feigenson GW. Phase Diagram of a Polyunsaturated Lipid Mixture: Brain Sphingomyelin/1-Stearoyl-2-Docosahexaenoyl-Sn-Glycero-3-Phosphocholine/Cholesterol. BBA-Biomembranes. 2016;1858(1):153–161.
